# Microcephaly protein ANKLE2 promotes Zika virus replication

**DOI:** 10.1128/mbio.02683-24

**Published:** 2025-01-13

**Authors:** Adam T. Fishburn, Cole J. Florio, Thomas N. Klaessens, Brian Prince, Neil A. B. Adia, Nicholas J. Lopez, Nitin Sai Beesabathuni, Sydney S. Becker, Liubov Cherkashchenko, Sophia T. Haggard Arcé, Vivian Hoang, Traci N. Shiu, R. Blake Richardson, Matthew J. Evans, Claudia Rückert, Priya S. Shah

**Affiliations:** 1 Department of Microbiology and Molecular Genetics, University of California, Davis, California, USA; 2 Department of Biochemistry and Molecular Biology, University of Nevada, Reno, Nevada, USA; 3 Department of Chemical Engineering, University of California, Davis, California, USA; 4 Department of Microbiology, Icahn School of Medicine at Mount Sinai, New York, New York, USA; Griffith University-Gold Coast Campus, Gold Coast, Queensland, Australia

**Keywords:** Zika virus, orthoflavivirus, NS4A, ANKLE2, virus-host interaction, microcephaly

## Abstract

Orthoflaviviruses are positive-sense single-stranded RNA viruses that hijack host proteins to promote their own replication. Zika virus (ZIKV) is infamous among orthoflaviviruses for its association with severe congenital birth defects, notably microcephaly. We previously mapped ZIKV-host protein interactions and identified the interaction between ZIKV non-structural protein 4A (NS4A) and host microcephaly protein ankyrin repeat and LEM domain-containing 2 (ANKLE2). Using a fruit fly model, we showed that NS4A induced microcephaly in an ANKLE2-dependent manner. Here, we explore the role of ANKLE2 in ZIKV replication to understand the biological significance of the interaction from a viral perspective. We observe that ANKLE2 localization is drastically shifted to sites of NS4A accumulation during infection and that knockout of ANKLE2 reduces ZIKV replication in multiple human cell lines. This decrease in virus replication is coupled with a moderate increase in innate immune activation. Using microscopy, we observe dysregulated formation of virus-induced endoplasmic reticulum rearrangements in ANKLE2 knockout cells. Knockdown of the ANKLE2 ortholog in *Aedes aegypti* cells also decreases virus replication, suggesting ANKLE2 is a beneficial replication factor across hosts. Finally, we show that NS4A from four other orthoflaviviruses physically interacts with ANKLE2 and is also beneficial to their replication. Thus, ANKLE2 likely promotes orthoflavivirus replication by regulating membrane rearrangements that serve to accelerate viral genome replication and protect viral dsRNA from immune detection. Taken together with our previous results, our findings indicate that ZIKV and other orthoflaviviruses hijack ANKLE2 for a conserved role in replication, and this drives unique pathogenesis for ZIKV since ANKLE2 has essential roles in developing tissues.

**IMPORTANCE:**

ZIKV is a major concern due to its association with birth defects, including microcephaly. We previously identified a physical interaction between ZIKV NS4A and host microcephaly protein ANKLE2. Mutations in ANKLE2 cause congenital microcephaly, and NS4A induces microcephaly in an ANKLE2-dependent manner. Here, we establish the role of ANKLE2 in ZIKV replication. Depletion of ANKLE2 from cells significantly reduces ZIKV replication and disrupts virus-induced membrane rearrangements. ANKLE2’s ability to promote ZIKV replication is conserved in mosquito cells and for other related mosquito-borne orthoflaviviruses. Our data point to an overall model in which ANKLE2 regulates virus-induced membrane rearrangements to accelerate orthoflavivirus replication and avoid immune detection. However, ANKLE2’s unique role in ZIKV NS4A-induced microcephaly is a consequence of ZIKV infection of important developing tissues in which ANKLE2 has essential roles.

## INTRODUCTION

Orthoflaviviruses are positive-sense single-stranded RNA viruses that cause severe disease. Many orthoflaviviruses, such as Zika virus (ZIKV), dengue virus (DENV), West Nile virus (WNV), and yellow fever virus (YFV), are transmitted by mosquitoes and represent significant public health threats worldwide. The RNA genome of orthoflaviviruses is roughly ~11 kb. Upon entry, this genome is directly translated into a single viral polyprotein and cleaved by host and viral proteases into 10 individual viral proteins. Three structural proteins (capsid, prM, and envelope [E]) make up the physical virion, while seven non-structural (NS) proteins (NS1, NS2A, NS2B, NS3, non-structural protein 4A [NS4A], NS4B, and NS5) facilitate aspects of virus replication within the host cell (1). Orthoflavivirus replication occurs on the cytoplasmic side of the host endoplasmic reticulum (ER) membrane, which is remodeled by other viral proteins to form virus replication organelles (2, 3). These organelles include replication compartments or vesicles (Ve), which bud into the ER membrane to concentrate substrates and conceal newly generated dsRNA from host detection (4). In addition to Ve, orthoflaviviruses also generate amorphous structures called convoluted membranes (CM). CM are believed to mediate aspects of protein translation, maturation, and degradation (5–7). NS4A is integral to the formation of orthoflavivirus replication organelles in the ER by inducing membrane curvature (8, 9) and through its interactions with host proteins (10–13).

ZIKV emerged as a global threat in 2015 during an epidemic that spread across South and Central America (14). ZIKV infection in adults typically leads to mild symptoms and very rarely Guillain-Barré syndrome (15). The primary concern surrounding ZIKV arises from the occurrence of congenital Zika syndrome (CZS) in individuals infected *in utero* (16). CZS is a spectrum of disease and can be clinically characterized by multiple hallmark features, including congenital contractures, ocular anomalies, cortical calcifications, and in the most severe cases, microcephaly (17, 18). Microcephaly is a condition in which the head and brain size are significantly reduced at birth (more than two standard deviations below the mean), and it is associated with a wide range of complications, including developmental delays, intellectual disability, and predisposition to seizures (19). In recent years, significant research has been dedicated to understanding mechanisms of ZIKV-induced neuropathogenesis. The mechanisms can be non-specific and broadly acting, such as the cytopathic effect of virus replicating in developing tissues, the systemic immune response, and the disruption of important biological barriers (e.g., placenta and blood-brain barrier) (20, 21). Mechanisms can also be highly specific, such as modulation of neuronal or neural progenitor cell growth through molecular interactions between viral and host components (22–24).

Previously, we used affinity purification and mass spectrometry to identify ZIKV-host protein-protein interactions that may contribute to the development of microcephaly in CZS (25). By searching for host proteins with known roles in neurodevelopment or associations with microcephaly, we identified the interaction between ZIKV NS4A and host ankyrin repeat and LEM domain-containing 2 (ANKLE2). ANKLE2 is primarily considered a scaffolding protein, facilitating protein-protein interactions between kinases, phosphatases, and their substrates (26). ANKLE2 localizes to the ER and inner nuclear membrane, where it mediates interactions with proteins, including barrier to autointegration factor (BANF1), vaccinia related kinase 1 (VRK1), and protein phosphatase complex 2A (PP2A), to control nuclear membrane disassembly during cell division (27, 28). Pathogenic mutations in *ANKLE2* cause congenital microcephaly in humans (29–31). Loss-of-function mutations in fly *Ankle2* cause small brain phenotypes and cellular defects in neuroblasts of third instar larvae. These phenotypes are rescued by the expression of human ANKLE2, suggesting that human ANKLE2 and fly Ankle2 are functionally conserved in brain development (29, 31). Using this *Drosophila* model, we previously showed that transgenic expression of ZIKV NS4A induces similar microcephaly phenotypes which are also rescued by the expression of human ANKLE2. Overall, this suggests NS4A induces microcephaly *in vivo* in an ANKLE2-dependent manner (25, 29).

Whether NS4A inhibition of ANKLE2 function during development is simply an unfortunate coincidence or if there is a functional role for this virus-host protein interaction in ZIKV replication is unknown. In this study, we explore the possibility that ANKLE2 plays a role in ZIKV replication. We find ANKLE2 concentrates at sites of NS4A accumulation during infection. Depletion of ANKLE2 reduces ZIKV replication in multiple cell lines that represent biologically relevant sites of ZIKV infection. This is accompanied by a moderate increase in innate immune response gene expression. Transmission electron microscopy (TEM) of ANKLE2 knockout (KO) cells reveals deficiencies in virus-induced membrane rearrangements, supporting a model in which ANKLE2 likely promotes ER remodeling to simultaneously accelerate genome replication and conceal viral dsRNA from host detection. Furthermore, we show that silencing of the ANKLE2 ortholog in mosquito cells also reduces ZIKV replication, suggesting a conserved role in replication across hosts. The NS4A-ANKLE2 interaction is conserved across mosquito-borne orthoflaviviruses, and ANKLE2’s role in virus replication is conserved to varying degrees. Altogether, we report the novel function of ANKLE2 in promoting ZIKV infection, providing evidence that NS4A disruption of neurodevelopment through ANKLE2 may arise from a general underlying mechanism of orthoflavivirus replication.

## RESULTS

### ANKLE2 co-localizes with ZIKV proteins during infection

**Fig 1:**
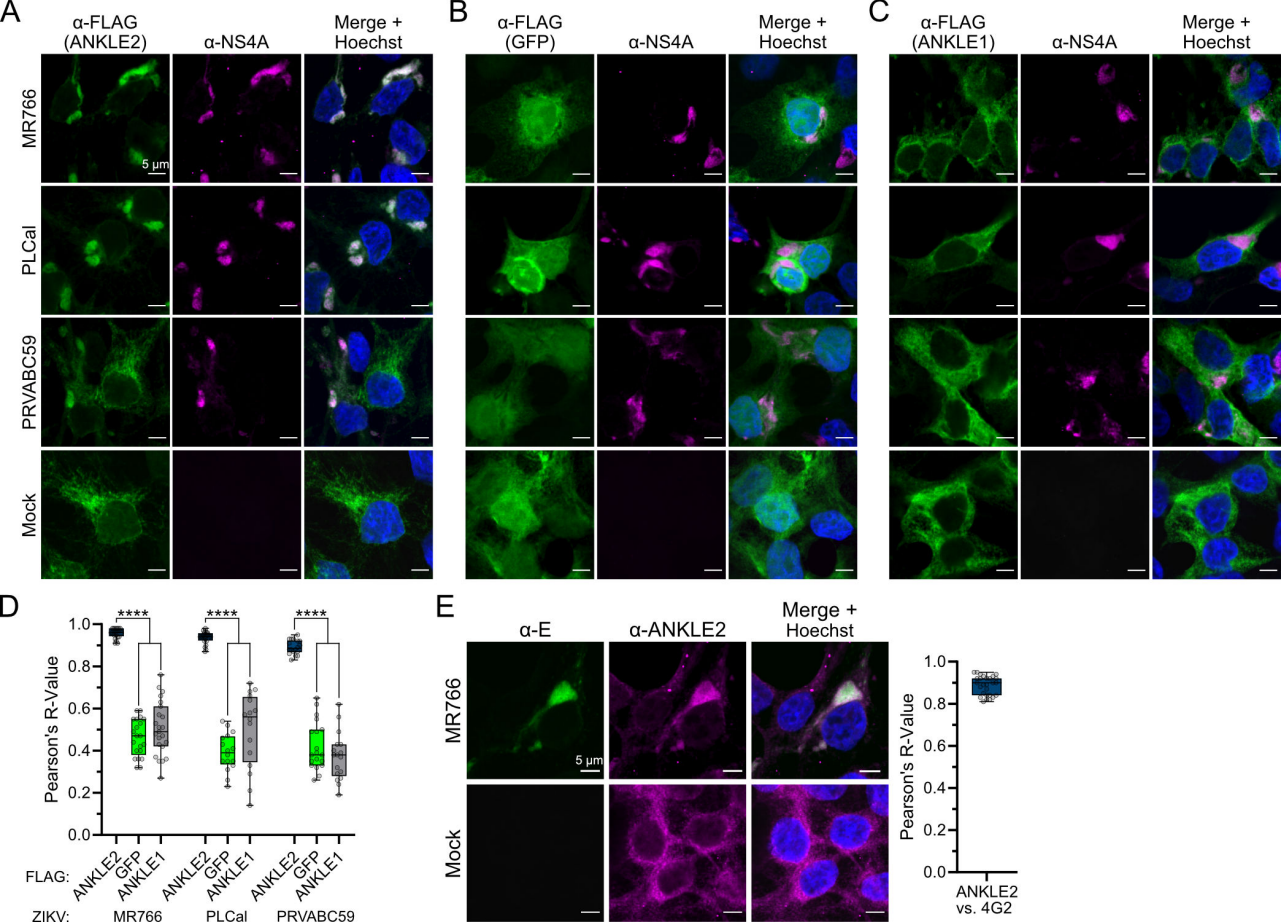
ANKLE2 co-localizes with ZIKV proteins during infection. (**A–C**) Immunofluorescence confocal microscopy of HEK293T cells expressing either ANKLE2-, GFP-, or ANKLE1-FLAG and infected with designated ZIKV strain at a multiplicity of infection (MOI) of 5 for 48 hours. Nucleus observed via Hoechst 33342 staining. (**D**) Pearson’s correlation was used to quantify degree of co-localization between FLAG and NS4A, *n* = 16–24 cells per condition. ****P < 0.0001, one-way analysis of variance with Šidák multiple comparison test. Gray circles represent the values of individual cells. (**E**) Microscopy of HEK293T cells infected with ZIKV MR766 at an MOI of 5 for 48 hours and evaluated for ZIKV E and endogenous ANKLE2, *n* = 27 cells. All scale bars = 5 µm.

Our previous studies established the interaction between ANKLE2 and NS4A (25) but only in the context of exogenously expressing a single viral protein. Therefore, we evaluated ANKLE2 subcellular localization during ZIKV infection. We generated HEK293T cells that express FLAG affinity tagged fusions of ANKLE2. As controls, we also generated cells that express GFP-FLAG as a general non-specific control and ANKLE1-FLAG to distinguish between intrinsic localization in this family of proteins and localization unique to ANKLE2. We infected these cells with three ZIKV strains (PLCal, MR766, and PRVABC59) and evaluated the co-localization of FLAG and NS4A. Strikingly, we found that ANKLE2 distribution became concentrated after infection, with near-perfect overlap with NS4A (Fig. 1A). Conversely, we found that the localization of GFP and ANKLE1 did not change following ZIKV infection (Fig. 1B and C). We measured co-localization between FLAG and NS4A signal using Pearson’s correlation and consistently found very high levels of co-localization between ANKLE2 and NS4A, which was significantly higher than GFP or ANKLE1 with NS4A (Fig. 1D). We also observed similar patterns when we performed immunofluorescence to compare endogenous ANKLE2 against ZIKV E protein (Fig. 1E). Given the tight co-localization between ANKLE2 and multiple ZIKV proteins, we hypothesized that ANKLE2 may play a role in virus replication. From these experiments, we also observed that HEK293T permissiveness to ZIKV was low overall and variable by ZIKV strain, as others have also shown (32). Therefore, we opted to use alternative cell lines and more contemporary ZIKV strains for subsequent experiments.

### ANKLE2 promotes ZIKV replication in multiple cell lines

We tested a potential role of ANKLE2 in ZIKV replication using a gene perturbation approach. We chose Huh7 cells, derived from a human hepatocarcinoma, since they are readily infected by ZIKV *in vitro* (33, 34), and the liver is an established site of ZIKV replication *in vivo* (35–37). Initially, we used a transient clustered regularly interspaced short palindromic repeats (CRISPR) interference (CRISPRi) knockdown system by reverse-transfecting guide RNAs (gRNAs) into dCas9-expressing Huh7 cells. We observed modest, but consistent, decreases in ZIKV replication following ANKLE2 knockdown across two ZIKV strains and a range of multiplicities of infection (MOIs) (Fig. S1). Importantly, impacts on cell viability were minor following ANKLE2 depletion (Fig. S1F). We hypothesized that these modest phenotypes arise from incomplete depletion of ANKLE2 in this system (Fig. S1C and H).

**Fig 2:**
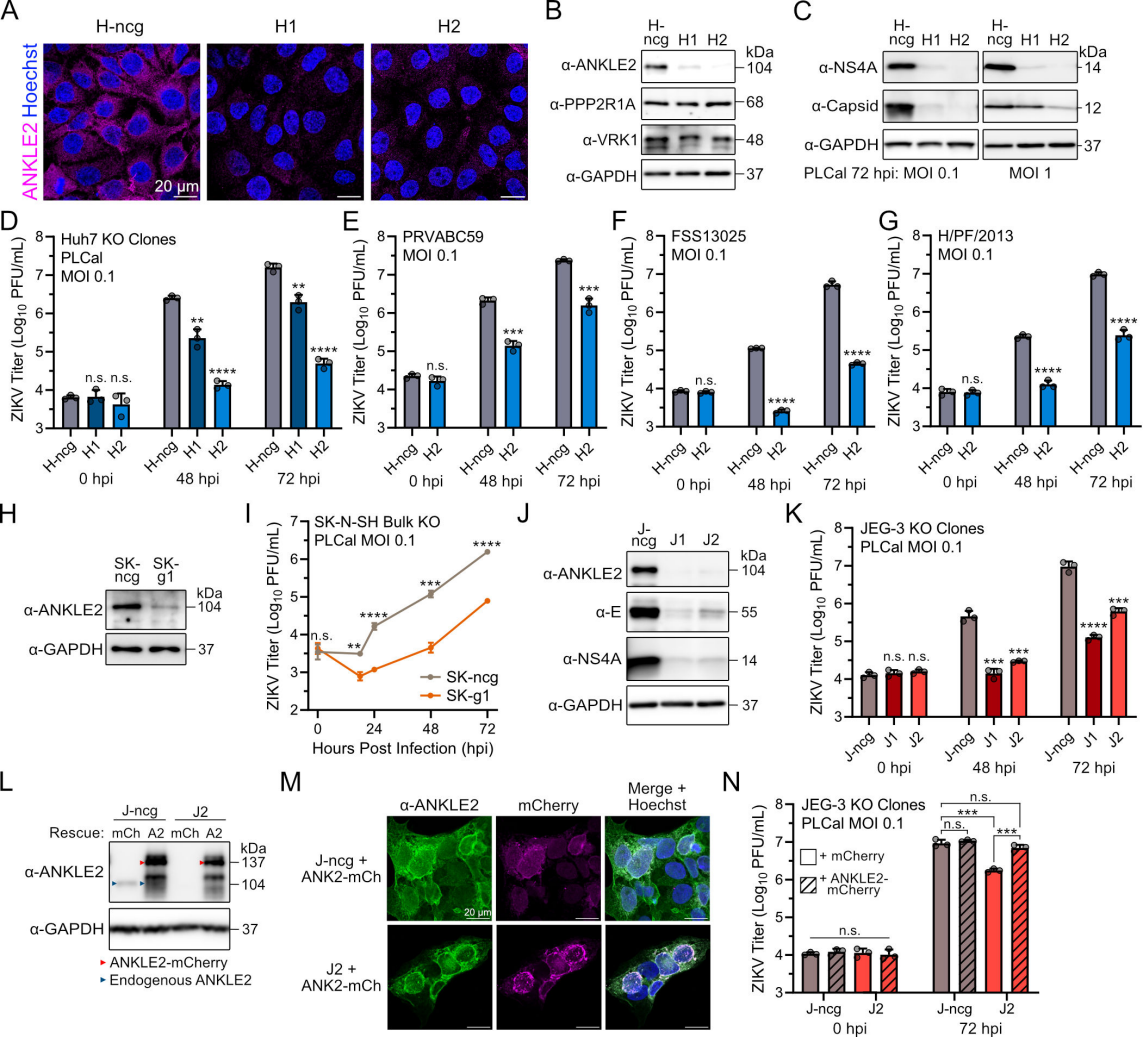
Knockout of ANKLE2 reduces ZIKV replication *in vitro* across multiple human cell lines. (**A**) Control Huh7 cells (H-ncg) and clonal ANKLE2 KO Huh7 cells (H1 and H2) were generated with CRISPR and validated by immunofluorescence confocal microscopy of ANKLE2. (**B**) Western blot of ANKLE2 and related proteins PPP2R1A and VRK1 in control and ANKLE2 KO cells. (**C**) Western blot of viral proteins following ZIKV PLCal infection of control and ANKLE2 KO cells. (**D–G**) Virus titer was determined by plaque assay following infection of control and ANKLE2 KO cells. All statistical comparisons are made to negative control (H-ncg) cells. (**H**) Western blot of control (SK-ncg) and ANKLE2 KO (SK-g1) SK-N-SH cells. (**I**) Virus titers measured by plaque assay following infection of control and ANKLE2 KO cells. (**J**) Western blot of control (J-ncg) and clonal ANKLE2 knockout (J1 and J2) JEG-3 cells following infection with ZIKV PLCal at an MOI of 0.1. (**K**) Virus titers measured by plaque assay following infection of control and ANKLE2 KO cells. (**L**) Western blot of JEG-3 cells transduced with mCherry (mCh) or ANKLE2-mCherry-3×FLAG (A2) lentivirus to overexpress or restore ANKLE2 expression. (**M**) Confocal microscopy of A2 rescue cells. (**N**) Virus titers measured by plaque assay from rescue cells infected with ZIKV PLCal at an MOI of 0.1 for 72 hours. Student’s two-tailed *t*-test: n.s., not significant; **P < 0.01, ***P < 0.001, ****P < 0.0001. Bars represent average values + standard deviations of three technical replicates.

We therefore sought to achieve a more complete depletion of ANKLE2 in Huh7 cells using CRISPR mutagenesis. We generated two stable KO clones by targeting the first exon of *ANKLE2* (hereon referred to as H1 and H2). As a control, we also generated a bulk cell line using a non-specific negative control gRNA (referred to as H-ncg). By immunofluorescence, we observed substantial depletion of ANKLE2 in our two clonal populations with some background signal (Fig. 2A; Fig. S2A). By Western blot, we again observed this depletion in both clones, with some residual ANKLE2 remaining in the H1 clone. We also observed no expression changes in the canonical ANKLE2-interacting proteins PPP2R1A (subunit of PP2A complex) and VRK1 (Fig. 2B), which facilitate nuclear membrane dynamics, in part through their interaction with ANKLE2 (26, 27). We evaluated virus replication in these cells by infecting them with ZIKV PLCal at an MOI of 0.1 and 1.0. The abundance of ZIKV proteins (NS4A and capsid) was strongly reduced in our clonal populations compared to the H-ncg control at 72 hours post-infection (hpi) (Fig. 2C). Next, we measured virus titers and found significant and consistent reduction at three MOIs (0.1, 1.0, and 10.0), with a maximum decrease of ~2.5 logs in the H2 clone when infected at an MOI of 0.1 (Fig. 2D; Fig. S2B and C). Interestingly, we observed a consistent difference between clones H1 and H2, with H2 having the more dramatic phenotype. This is supported by a small amount of ANKLE2 remaining in the H1 clone (Fig. 2B). The more modest phenotype in our H1 clone resembles the ~1 log decreases in virus titer that we observed in CRISPRi experiments where ANKLE2 expression was only partially suppressed (Fig. S1). Furthermore, DNA sequencing our clones revealed H2 had a single *ANKLE2* mutation, suggesting they are a monoclonal population, while H1 appeared to be a polyclonal population with four equally abundant mutations (Table S1). To validate our results, we infected H-ncg and H2 with other Asian lineage ZIKV strains, PRVABC59, FSS13025, or H/PF/2013, and observed similar ~1 to 2 log decreases in virus replication at 48 and 72 hpi (Fig. 2E through G).

To further characterize and validate our Huh7 clones, we performed high-throughput fluorescence microscopy, either in mock-, PLCal-, or PRVABC59-infected cells at an MOI of 1 for 48 hours. Previous studies showed ANKLE2 KO HeLa cells have dramatic increases in nuclear area (28); however, we did not observe any biologically relevant increase in our Huh7 KO cells (Fig. S2D). We also measured no difference in the number of mitotic cells in either clone, in the absence or presence of virus infection (Fig. S2E), and measured no differences in cell viability (Fig. S2F). Evaluating the percentage of infected cells recapitulated the results of our previous titration experiments, with decreases in H1 and H2 for both PLCal and PRVABC59 strains (Fig. S2G and H).

Finally, we sought to validate the specificity of our ANKLE2 KO phenotypes by restoring ANKLE2 expression to our KO cells. We transduced our H-ncg or H2 cells with lentivirus to stably induce expression of ANKLE2-mCherry-3×FLAG (A2) or mCherry (mCh) alone as a control. Confocal microscopy showed expression of our fusion protein and that expression in rescued cells was higher than endogenous levels (Fig. S3A). The fusion protein also retained correct ER localization (Fig. S3B and C). ZIKV infection of these cells revealed nearly complete absence of ZIKV E protein in H2 + mCh cells, with a moderate increase in H2 + A2 cells; however, this was still much less than either H-ncg population, suggesting only a partial rescue of the replication phenotype (Fig. S3D). This was consistent with virus titers which showed a ~0.5 log increase in our rescue population compared to the KO (H2 + A2 vs H2 + mCh) (Fig. S3E). Immunofluorescence microscopy revealed a rescue rate of only ~8%, suggesting that our partial rescue is due to incomplete ANKLE2 restoration (Fig. S3F). We measured the infection rate by immunofluorescence of this same cell population and observed a partial rescue of this infection rate, similar to our virus titer measurements (Fig. S3G). In fact, Fisher’s exact test revealed that the small number of successfully rescued H2 cells were significantly more likely to be infected than those without rescue (*P* value = 0.0003) (Fig. S3H). Altogether, these results suggest that our rescue phenotypes would be stronger if ANKLE2 expression were more homogeneously restored and that ANKLE2 supports ZIKV infection in Huh7 cells.

ANKLE2 is broadly expressed in a variety of tissues throughout development and may have tissue-specific functions. To establish if ANKLE2 has tissue-specific roles in ZIKV replication, we subsequently tested cell lines representative of tissues targeted by ZIKV during human infection. ZIKV replicates in the developing brain, and we therefore examined the role of ANKLE2 in neuroblastoma SK-N-SH cells. We observed a similar overlap between ZIKV proteins and ANKLE2 in these cells (Fig. S2I). We generated a bulk KO population (SK-g1), with an accompanying control line (SK-ncg) (Fig. 2H). Unfortunately, SK-N-SH cells were difficult to grow at low cell densities in our hands, so we were unable to generate clonal KO populations. Regardless, ZIKV infection in SK-g1 cells revealed substantial reduction of virus titers (Fig. 2I).

The placenta is also actively infected during human ZIKV infection, and this process is very likely responsible for vertical transmission (38, 39). We therefore generated additional KO clones in placental choriocarcinoma JEG-3 cells (referred to as J1 and J2) with an accompanying control line (J-ncg). ZIKV-infected cells displayed a complete depletion of ANKLE2 and reduced levels of ZIKV E and NS4A in J1 and J2 cells (Fig. 2J). We also observed a strong ~1 to 2 log reduction in virus titers in J1 and J2 cells compared to J-ncg (Fig. 2K). Finally, we sought to rescue replication phenotypes by restoring ANKLE2 expression in JEG-3 cells. As done previously, we transduced our J-ncg or J2 cells with lentivirus to stably induce expression of ANKLE2-mCherry-3×FLAG (A2) (Fig. 2L and M) or mCh (not shown). J2 + A2 cells had similar virus titers to J-ncg cells and significantly more than J2 + mCh (Fig. 2N). We did not observe any further increase in E protein or virus titers from J-ncg + mCh to J-ncg + A2 cells, suggesting that overexpression of ANKLE2 does not further increase ZIKV replication. Taken together, we conclude that ANKLE2 supports ZIKV replication in multiple human cell types.

### ANKLE2 promotes ZIKV genome replication and reduces innate immune activation

**Fig 3:**
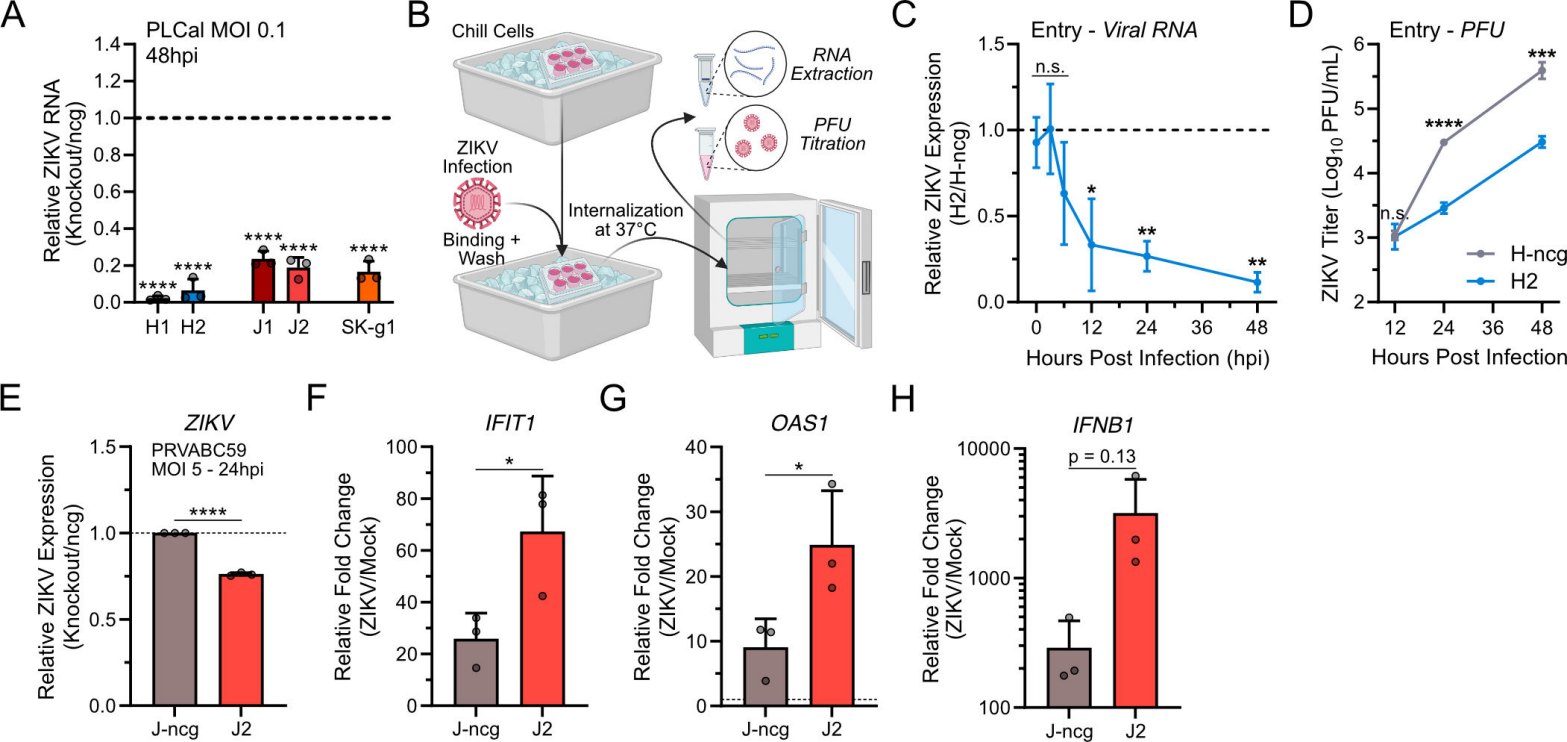
Knockout of ANKLE2 reduces ZIKV genome replication and enhances innate immune response activation. (**A**) RT-qPCR to assess ZIKV in ANKLE2 KO Huh7, JEG-3, and SK-N-SH cells infected with ZIKV PLCal at an MOI of 0.1 for 48 hours. Student’s unpaired *t*-test compared to corresponding normalized ncg control (dashed line). (**B–D**) Entry and internalization assay was performed after incubation with ZIKV PRVABC59 at an MOI of 2. RNA or virus titers were evaluated by RT-qPCR (**C**) or plaque assay (**D**). (**E–H**) RT-qPCR to assess innate immune responses in cells infected with ZIKV PRVABC59 at an MOI of 5 for 24 hours. Relative expression shown compared to the same cell line under mock infection conditions. Student’s unpaired *t*-test: n.s., not significant; *P < 0.05, **P < 0.01, ***P < 0.001, ****P < 0.0001. Bars represent average values + standard deviations of three biological replicates.

Next, we sought to determine the mechanism by which ANKLE2 promotes ZIKV replication. As a complementary approach to our previous experiments, we first evaluated the fold change of viral genomic material using reverse transcriptase-PCR (RT-qPCR). Here, we observed ~80% to 90% decreases in all our ANKLE2 KO cell lines, suggesting genome replication may be impacted (Fig. 3A). To identify the point in the virus replication cycle contributing to this effect, we used a synchronized ZIKV binding and entry assay to measure virion binding, internalization, genome replication, and infectious virion production (Fig. 3B) (40, 41). This experiment revealed no differences between H-ncg and H2 cells in virion binding at time 0 or early virion internalization at 3 hpi, suggesting ANKLE2 is not involved in the entry process since this process occurs on the order of minutes for orthoflaviviruses (42, 43). We did observe decreased viral genome production in H2 cells beginning at 12 hpi and becoming more substantial over time (Fig. 3C). This manifested in a reduction in virion production beginning at 24 hpi (Fig. 3D). Together, these data suggest that ANKLE2 promotes early virus genome replication prior to virus egress and spread.

Next, we sought to identify the molecular mechanism by which ANKLE2 promotes ZIKV replication. We explored autophagy since it is implicated in orthoflavivirus replication (44, 45) but did not observe any appreciable difference in LC3B I or II abundance (Fig. S4A). Since ANKLE2 is present in the ER membrane where ZIKV polypeptide processing occurs, we explored if KO cells were deficient in signal peptidase cleavage. To test this, we expressed 2K-NS4B in Huh7 control and KO cells but again did not observe any differences in 2K-NS4B cleavage (Fig. S4B). Finally, we aimed to investigate differences in immune activation and ER stress responses. We infected at an MOI of 5 to ensure a robust response and evaluated gene expression at 24 hpi. At this high MOI, we found that ZIKV replication was more modestly decreased in J2 KO cells compared to the control (Fig. 3E), similar to our results at high MOI in Huh7 cells earlier (Fig. S2C). Despite the decrease in virus replication in J2 cells, we observed a significant increase in expression of *IFIT1* and *OAS1* (Fig. 3F and G). Though the increase in *IFNB1* gene expression was substantial (10-fold more induction than control cells), the change was not statistically significant (Fig. 3H). The J1 clone showed similar trends in immune gene expression but with more variability (Fig. S4C). We did not observe any changes in the induction of ER-stress response genes *XBP1s* or *ATF6* after infection (Fig. S4D) or treatment with tunicamycin (Fig. S4E). Together with our previous data, we conclude that ANKLE2 supports early ZIKV genome replication and may contribute to early ZIKV immune evasion.

### ANKLE2 regulates ZIKV-induced ER rearrangements

**Fig 4:**
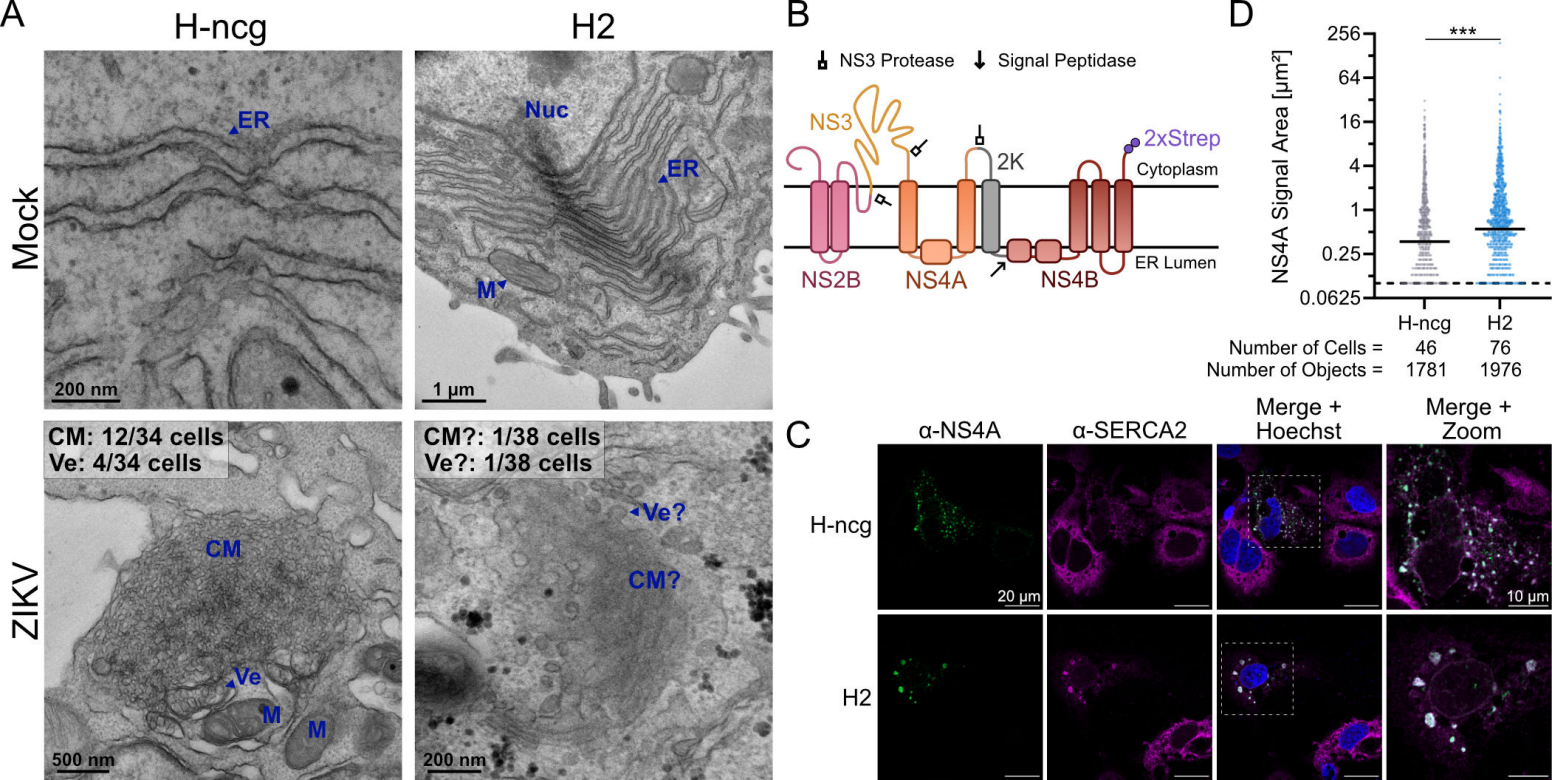
ZIKV membrane rearrangements are dysregulated in ANKLE2 knockout Huh7 cells. (**A**) TEM images of cells that were either mock-infected (top) or infected with ZIKV PRVABC59 at an MOI of 1 for 48 hours (bottom). Stereotypical orthoflavivirus replication organelles were consistently observed in infected H-ncg cells, including virus-induced replication vesicles (Ve) and convoluted membranes (CM). The number of cells with observed CM and Ve are noted in the top left corners. Other abbreviations: ER, endoplasmic reticulum, M, mitochondrion, Nuc, nucleus. (**B–D**) H-ncg or H2 cells were transfected with a plasmid expressing ZIKV NS2B-3-4A-2K-4B for 48 hours. Induced membrane rearrangements were evaluated by confocal microscopy (**C**) or quantified using a semi-automated imaging pipeline (**D**). Data represent four biological replicates. Black lines indicate median NS4A aggregate size; dashed line indicates limit of detection set to ≥4 pixels of signal. Student’s unpaired *t*-test: ***P < 0.001.

Orthoflaviviruses create virus replication organelles through massive ER membrane rearrangements. These virus replication organelles are hypothesized to accelerate viral genome replication and provide a hiding spot for dsRNA to avoid immune detection. The increased immune gene expression despite lower ZIKV genome replication following ANKLE2 KO prompted us to consider ANKLE2’s role in ZIKV-induced membrane rearrangements. To explore this, we examined the structure of the ER and virus replication organelles in control H-ncg and H2 cells by TEM (Fig. 4A). The ER in mock-infected cells appeared normal in both H-ncg and H2 cells, suggesting that ANKLE2 is not inherently involved in higher-level ER integrity, organization, or structure. In ZIKV-infected H-ncg cells, we observed stereotypical virus replication organelles in 12 out of 34 cells analyzed, including clusters of CM and Ve matching the expected size and organization (2). In ZIKV-infected H2 cells, we only observed replication organelles in 1 out of 38 cells, and the structure of CM was poorly defined, suggesting that ER rearrangements might be dysregulated in ANKLE2 KO cells. However, the lack of CM in these cells may arise simply due to delayed replication kinetics. To test our hypothesis outside the context of infection, we generated a plasmid to express four non-structural proteins from ZIKV PRVABC59 (Fig. 4B). These viral proteins are sufficient to induce membrane rearrangements *in vitro* (9). Following transfection into H-ncg and H2 cells, we confirmed that aggregations of NS4A protein are co-localized with the ER-marker SERCA2. Moreover, we observed larger aggregations in H2 cells (Fig. 4C). To confirm this, we used a semi-automated high-throughput imaging pipeline to count and quantify the size of NS4A aggregates (Fig. 4D). From this experiment, we found that NS4A aggregates were ~1.9 times larger in H2 cells than H-ncg cells. Together, we conclude that ANKLE2 likely mediates some aspects of ER rearrangements during ZIKV infection, the dysregulation of which may drive increased immune induction and decreased ZIKV replication.

### Mosquito ANKLE2 promotes ZIKV replication

**Fig 5:**
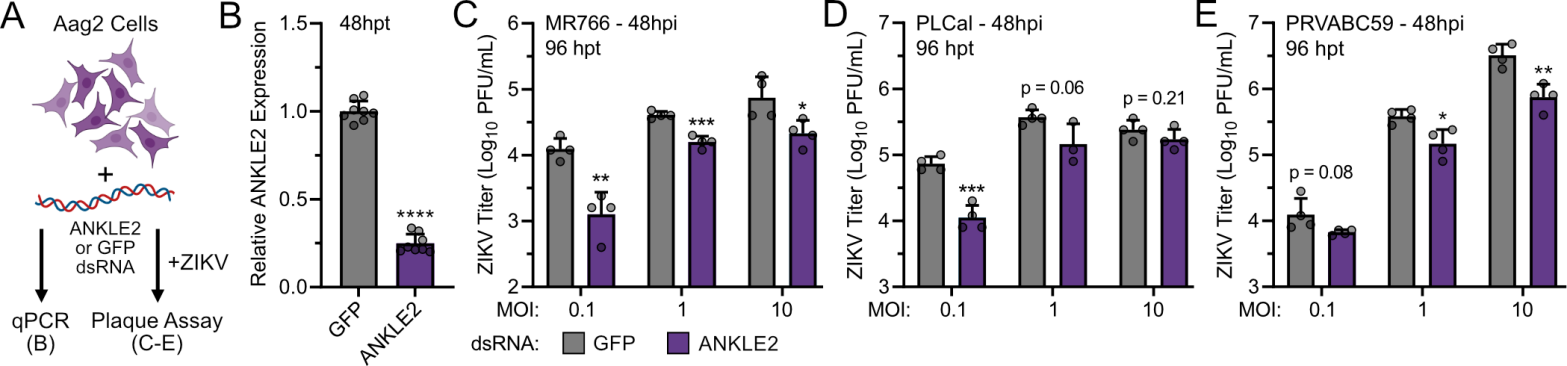
Silencing of ANKLE2 ortholog in mosquito Aag2 cells reduces ZIKV replication. (**A**) Mosquito Aag2 cells were transfected with either GFP- or ANKLE2 (LOC5576059, UniProtID A0A6I8U7J4) ortholog-targeting dsRNA for 48 hours prior to RT-qPCR or ZIKV infection. (**B**) ANKLE2 ortholog expression was measured by RT-qPCR. Eight technical replicates across two biological experiments. (**C–E**) Virus titers were measured by plaque assay for GFP and ANKLE2 ortholog knockdown cells for the noted ZIKV strain and MOI. Four knockdown/infection technical replicates were performed for each condition. All statistical comparisons are made to the corresponding GFP condition. Student’s unpaired *t*-test: *P < 0.05, **P < 0.01, ***P < 0.001, ****P < 0.0001.

While ZIKV can be transmitted between humans sexually, vertically, and via blood or organ donation, it is primarily an arbovirus transmitted by *Aedes* mosquitoes. Successful transmission requires active replication in the mosquito vector. Given that ZIKV benefits from ANKLE2 in human cells, we hypothesized that ZIKV similarly uses *Aedes aegypti* ANKLE2 in some capacity as a conserved host replication factor. To test this hypothesis, we performed dsRNA knockdown of *ANKLE2 in vitro* using Aag2 cells with a GFP targeting dsRNA as a negative control (Fig. 5A). Here, we achieved modest decreases in *ANKLE2* mRNA expression after 48 hours (Fig. 5B). We then infected these cells with three strains of ZIKV, at three MOIs each, and assessed virus titers by plaque assay. We observed mostly consistent decreases in ZIKV titer at 48 hpi across MOIs and ZIKV strains (Fig. 5C through E; Fig. S5), though incomplete or unsustained knockdown may limit the magnitude of the replication phenotype. These experiments support our hypothesis that ANKLE2 is a replication factor conserved across hosts.

### ANKLE2 interacts with and mediates replication of multiple orthoflaviviruses

**Fig 6:**
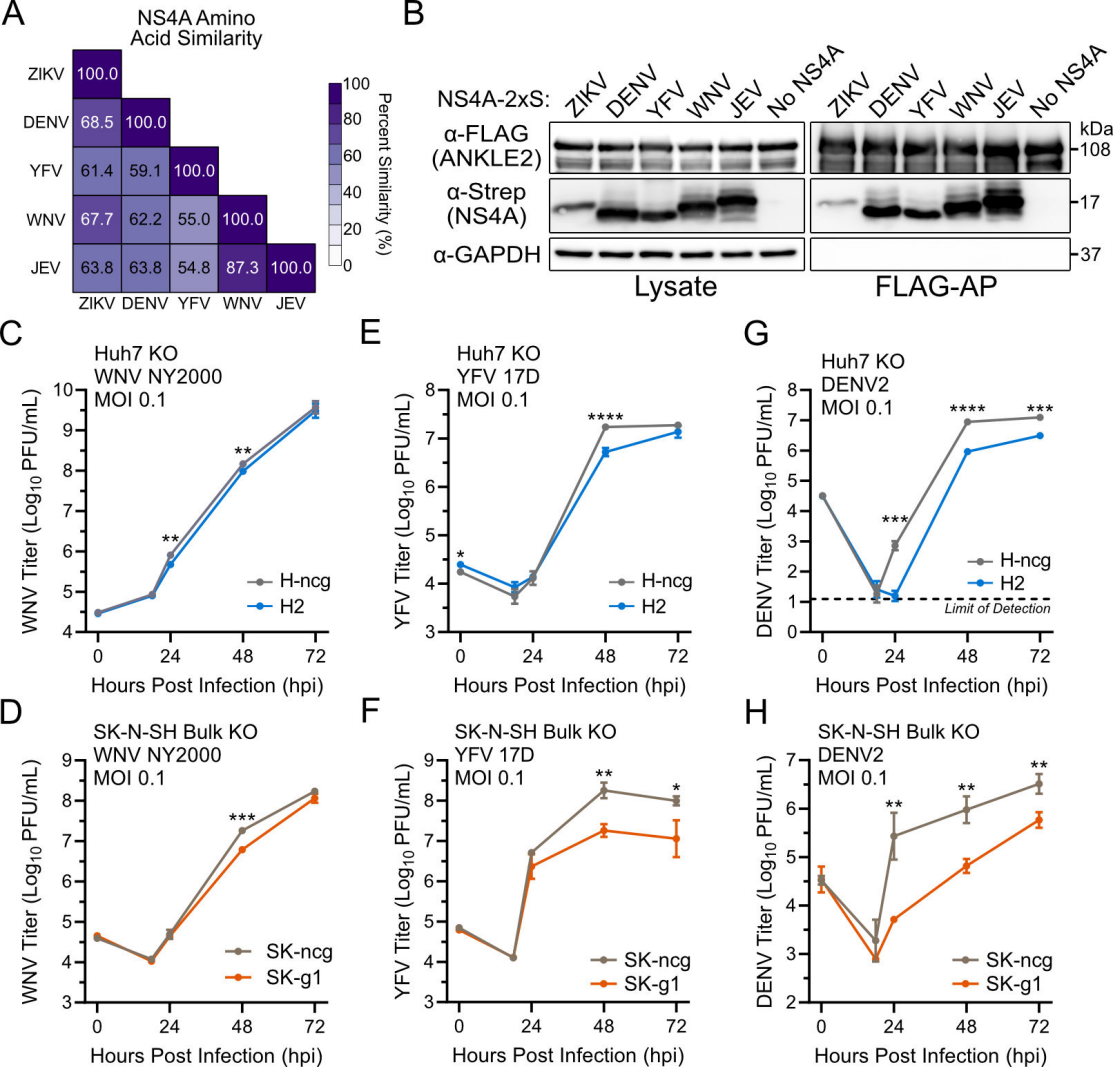
The NS4A-ANKLE2 interaction is conserved across mosquito-borne orthoflaviviruses, but the impact of ANKLE2 in virus replication varies. (**A**) NS4A total amino acid biochemical similarity using EMBOSS Needle (46). (**B**) HEK293T cells were co-transfected with ANKLE2-3×FLAG and NS4A-2×Strep from the corresponding orthoflavivirus. FLAG affinity purification (AP) and Western blotting were performed to determine physical interaction between proteins. (**C–H**) Huh7 or SK-N-SH ANKLE2 KO cells were infected with indicated viruses at an MOI of 0.1 for 72 hours. Virus titers were measured using plaque assay. All values shown are means ± standard deviations of three technical replicates. All statistical tests compared to corresponding ncgRNA condition. Student’s *t*-test: n.s., not significant. *P < 0.05, **P < 0.01, ***P < 0.001, ****P < 0.0001. ncg, negative control CRISPR gRNA control.

Our data support that ZIKV NS4A interacts with ANKLE2 (25) and that ANKLE2 promotes fundamental aspects of orthoflavivirus replication. We next sought to explore the conservation of both the physical interaction and role in replication for other mosquito-borne orthoflaviviruses. Comparing NS4A amino acid similarity showed moderately high conservation among four other commonly studied mosquito-borne orthoflaviviruses (Fig. 6A). We generated NS4A C-terminal strep-tagged fusions for these orthoflaviviruses (DENV, WNV, YFV, and Japanese encephalitis virus [JEV]) and evaluated their physical interaction with ANKLE2-3×FLAG using FLAG affinity purification and Western blotting. We observed that these NS4As expressed higher than ZIKV NS4A, and all co-purified with ANKLE2-3×FLAG (Fig. 6B). This suggests that the NS4A-ANKLE2 physical interaction is conserved across mosquito-borne orthoflaviviruses.

To evaluate a potential role for ANKLE2 in orthoflavivirus replication beyond ZIKV, we infected our Huh7 and SK-N-SH ANKLE2 KO cells with WNV (NY2000), YFV (17D), and DENV (serotype 2 16681). For WNV, we observed statistically significant but likely biologically irrelevant decreases in virus titers, since the magnitude of the decrease is less than half of a log (Fig. 6C and D). Infection with YFV resulted in a minor decrease at 48 hpi in H2 cells and a more substantial ~1 log decrease in SK-N-SH cells (Fig. 6E and F). DENV infection resulted in a large earlier replication deficit both cell types and sustained over 72 hours (Fig. 6G and H). Together, these data suggest that multiple orthoflaviviruses interact with ANKLE2, though its impact on orthoflavivirus replication *in vitro* is variable.

## DISCUSSION

In this study, we explore the relationship between ZIKV and host ANKLE2, with a focus on virus replication. ANKLE2 co-localizes with ZIKV NS4A during infection, and depletion of ANKLE2 in human cells leads to consistent reduction in ZIKV replication during early genome replication. Microscopy suggested that ANKLE2 KO results in fewer and poorly formed virus replication organelles and significantly altered membrane rearrangements. This finding, combined with our observations that ANKLE2 KO cells appear to have increased innate immune induction, supports a model in which ANKLE2 promotes virus-mediated ER remodeling to accelerate and conceal genome replication. Interestingly, dsRNA knockdown of the ANKLE2 ortholog in mosquito cells produces similar, albeit modest, decreases in ZIKV replication. Furthermore, we showed that the physical interaction between NS4A and ANKLE2 is conserved across four additional mosquito-borne orthoflaviviruses and that ANKLE2 promotes DENV and YFV replication. Thus, ANKLE2 is a conserved mosquito-borne orthoflavivirus host replication factor.

The conserved role of ANKLE2 in replication across hosts and orthoflaviviruses revises our model in which ZIKV NS4A inhibits ANKLE2 to cause microcephaly (25, 29). Though ANKLE2 has a very specific and non-redundant role during fetal brain development, it may have additional roles in cell physiology when expressed in other tissues (26). Our data suggest that ANKLE2 is hijacked by orthoflaviviruses in general to facilitate replication in many different tissues and hosts. This is supported by our findings that depletion of ANKLE2 from three distinct cell types and two host species all show reduction in ZIKV replication. Interestingly, we found that only partial ANKLE2 reduction was required in neuroblastoma SK-N-SH cells to cause a significant impact on virus replication. From this, we hypothesize that ZIKV replication is more sensitive to the amount of ANKLE2 in neuronal cells than others. Our study also revealed that NS4A from non-teratogenic orthoflaviviruses can physically interact with ANKLE2, although these viruses do not cause microcephaly in humans. We speculate these NS4As are capable of inhibiting ANKLE2 function through a conserved protein interaction, though this is inconsequential in tissues naturally targeted by these non-teratogenic orthoflaviviruses. If these orthoflaviviruses were able to gain access to the developing fetal brain, they also have the potential to cause ANKLE2-dependent microcephaly. This is supported by our previous data that show transgenic expression of DENV NS4A modestly inhibits brain development in larval fruit flies (25) but does not cause microcephaly in humans. Thus, ZIKV’s unique pathogenesis derived from the NS4A-ANKLE2 interactions is from being in the wrong place at the wrong time when it comes to ANKLE2.

It is interesting that while the NS4A-ANKLE2 interaction appears broadly conserved, the degree to which virus replication is impacted is variable across mosquito-borne orthoflaviviruses. While we observed strong impacts on replication for ZIKV and DENV, we observed a more modest effect on YFV and essentially no effect on WNV. We hypothesize that the interaction may have first evolved in an ancestral virus, and while the interaction is conserved, the impact on replication was lost. We speculate that this may explain the observed pattern, given that ZIKV, DENV, and YFV replicate in *Aedes* spp., while WNV is transmitted by *Culex* spp. Future experiments testing ANKLE2 in *Culex* spp. and for tick-borne orthoflaviviruses will help establish if there is an evolutionary basis for these differences. Alternatively, WNV may be less sensitive to ANKLE2 depletion *in vitro* or cell types tested since it readily replicates to high titers. Support for this comes from the fact that ANKLE2 KO replication phenotypes are anticorrelated with peak titers for WNV when comparing two different cell lines (Huh7 vs SK-N-SH). A cell line that less efficiently replicates WNV may show the potential benefit of ANKLE2 more readily.

The supportive role of ANKLE2 in ZIKV replication raises exciting possibilities about ANKLE2 function during orthoflavivirus replication. Beyond BANF1, PP2A, and VRK1 (27, 28), ANKLE2 also interacts with many other host proteins. For example, ANKLE2 also influences the cell cycle by interacting with Aurora-A and estrogen receptor alpha (ERα) to mediate ERα phosphorylation (47). Plentiful other ANKLE2-host protein interactions have been found in proteomic screens (48–51). It is particularly appealing to speculate that ZIKV leverages the protein interaction/scaffolding function of ANKLE2 to facilitate protein interactions within orthoflavivirus replication organelles. Orthoflavivirus non-structural proteins interact with ER-associated degradation factors, which regulate CM morphogenesis, virus propagation, and virus-induced cell death (7, 52, 53). DENV utilizes the host protein HMGCR in the process of replication complex formation, which is significant as HMGCR is also regulated by PP2A (54). Many other host factors are co-opted by orthoflaviviruses to remodel the ER and assist in the formation of replication compartments or complexes (10, 12, 55–59), and ANKLE2 may play a scaffolding role in this remodeling process. This is especially interesting, given the established roles of NS4A in facilitating orthoflavivirus replication organelle formation. Our study draws similarities to that of Hoffman et al., which showed ZIKV NS4A interacts with host TMEM41B to remodel ER membranes (58). Depletion of TMEM41B showed significant reduction in virus replication, increase in antiviral immune activation, and dysregulation of virus-induced organelle formation, a pattern similar to what we have observed with depletion of ANKLE2. Future studies exploring ANKLE2 protein interactions during orthoflavivirus infection will be valuable in uncovering the molecular mechanisms by which ANKLE2 accelerates orthoflavivirus replication.

It is important to note caveats related to our studies. Our data suggest a model in which ANKLE2 promotes the proper formation of replication organelles during infection, which accelerates viral genome replication and conceals viral dsRNA from immune sensing. However, this replication phenotype and our previously published pathogenesis (25, 29) phenotype may not necessarily be linked to each other or the protein interaction itself. It is also possible that ANKLE2 facilitates a separate function in immune regulation independent of its role in genome replication. The role of ANKLE2 in ZIKV replication and pathogenesis *in vivo* remains to be determined, although this is technically challenging since *Ankle2* is essential to embryonic mouse development. Additionally, in both our human and mosquito cell culture models, we observed variable effects on virus replication dependent on virus strain and MOI. We speculate that during high MOI infections, the overwhelming amount of virus can overcome the disadvantage posed by ANKLE2 depletion. Differential outcomes may also arise from underlying differences between ZIKV strains, outside of NS4A, that dictate replication dynamics. Finally, while depletion of ANKLE2 clearly impairs ZIKV replication, overexpression of ANKLE2 does not seem to enhance it further. Thus, there is an upper limit on how much ANKLE2 can promote formation of replication compartments, or there is another rate-limiting step in the virus replication cycle beyond ANKLE2.

To our knowledge, the NS4A-ANKLE2 protein interaction is a rare example of a ZIKV-host interaction that dysregulates the host function to cause neuropathogenesis (25, 29) in the process of promoting ZIKV replication. Several other protein interactions impact aspects of neurodevelopment but appear distinct from virus replication. ZIKV NS3 cleaves host BMP2, inducing osteogenesis and intracranial calcification commonly seen in CZS (22). Expression of NS2A *in vivo* impacts neurodevelopment by disrupting adherens junctions in radial glial cells (24). Expression of NS4A and NS4B impairs the growth of neural stem cells *in vitro* and perturbs autophagy (60). While the host factors from these studies are involved in pathogenesis, they were not linked to virus replication directly. A notable exception is the interaction between ZIKV capsid and Dicer. Capsid interacts with Dicer and inhibits its antiviral activity to promote ZIKV replication while simultaneously inducing neurodevelopmental defects (61). These types of virus-host interactions, which result in compounding losses for the host, represent an exciting system to simultaneously study virus replication and neuropathogenesis.

## MATERIALS AND METHODS

### Cells

HEK293T (gift of Dr. Sam Díaz-Muñoz), Huh7 (gift of Dr. Raul Andino), JEG-3 (American Type Culture Collection [ATCC]), SK-N-SH (ATCC), and Vero (ATCC) cell lines were maintained in Dulbecco’s modified Eagle’s medium (DMEM; Gibco, Thermo Fisher) supplemented with 10% fetal bovine serum (FBS; Gibco, Thermo Fisher) at 37°C, 5% CO_2_. Cells we washed with Dulbecco’s phosphate-buffered saline (D-PBS, Life Technologies) and dissociated with 0.05% trypsin-EDTA (Life Technologies). Cells were tested for *Mycoplasma* spp. monthly by PCR. Mosquito Aag2 (*Aedes aegypti*) cells were cultured at 27°C in Schneider’s *Drosophila* Medium (Gibco, +Glutamate) supplemented with 7% FBS and antibiotics (100 units mL penicillin, 100 µg/mL streptomycin, 5 µg/mL gentamicin). Cell viability was measured using a XTT viability assay kit according to manufacturer protocol (Biotium).

### Plasmids

Lentiviral plasmid (pHR-UCOE-EF1α-KRAB-dCas9-P2A-Bls) encoding a catalytically dead Cas9 (dCas9) with a C-terminal hemagglutinin tag was a gift from Dr. Sean Collins. For stable, inducible expression in HEK293T cells, ANKLE2 and GFP sequences were amplified by PCR and cloned into pLVX-TetOne-Puro, cut with EcoRI, using Gibson assembly. Codon-optimized ANKLE1 sequence was acquired from Twist BioSciences and similarly inserted into pLVX-TetOne-Puro, cut with EcoRI, with C-terminal APEX2 and 3×FLAG affinity tags. Orthoflavivirus (DENV, YFV, WNV, and JEV) NS4A sequences were acquired from Twist Bioscience and inserted into pcDNA4_TO, cut with BamHI and XhoI, with C-terminal 2×Strep tags using Gibson assembly. The plasmids for expressing ZIKV 2K-NS4B or ZIKV NS2B-3-4A-2K-4B were prepared using a similar strategy. ANKLE2-mCherry-3×FLAG-P2A-BLS rescue fusions were cloned into pHR-UCOE-EF1α-KRAB-dCas9-P2A-Bls, which had been cut with MluI and NotI to remove dCas9. Gene fragments were acquired from Twist BioSciences and inserted with Gibson Assembly. All plasmids were prepared in Stbl3 or DH5α using MiniPrep (Sigma-Aldrich) or MidiPrep (Macherey-Nagel) kits and verified using sequencing services provided by GeneWiz. All sequence accession numbers are available in Table S3. Primer sequences used for the generation of all our constructs are available in Table S4.

### Lentiviral packaging, transduction, and cell selection

Lentiviral packaging and transduction were performed as previously described (62) using the calcium phosphate protocol (63). In short, 3.5 µg of cloning product plasmid was transfected into HEK293T with lentiviral packaging plasmids including 1.8 µg pMDLg/p-RRE, 1.25 µg pCMV-VSV-g, and 1.5 µg pRSV-Rev. After 48 hours, lentivirus particles were collected, and cell debris was removed by centrifugation (Eppendorf centrifuge 5810 R, Rotor S-4-104, 94 × *g*, 5 minutes) and filtration through a 0.45 µm filter. The resulting lentiviral stocks were used to transduce target cells. Transduced cells were bulk selected for puromycin or blasticidin resistance (1 or 10 µg/mL, respectively; Thermo Fisher). A control lentiviral plasmid encoding GFP without a selection marker was used in tandem as a control to ensure efficient packaging, transduction, and selection.

### Viruses and stock preparation

All virus (ZIKV, DENV, and WNV) stocks were propagated in Vero cells and monitored for CPE. Supernatant was then harvested, and cell debris was removed by centrifugation (Eppendorf centrifuge 5810 R, Rotor S-4-104, 211 × *g*, 5 minutes, 4°C). Cleared supernatant was then distributed into 1 mL aliquots and frozen at −80°C. Each aliquot was only used once to prevent repetitive freeze-thaw. Aliquots were titered by plaque assay (method below). Strains used were ZIKV PLCal/2013 (gift of Dr. Richard Wozniak), ZIKV PRVABC59 (gift of Dr. Lark Coffey), ZIKV FSS13025 (gift of Dr. Helen Lazear), ZIKV H/PF/2013 (gift of Dr. Helen Lazear), ZIKV MR766 (BEI Resources, National Institute of Allergy and Infectious Diseases, National Institutes of Health, as part of the WRCEVA program: Zika Virus, MR 766, NR-50065), DENV2 16681 (25), YFV 17D (gift of Dr. Lark Coffey), and WNV NY2000 (gift of Dr. Helen Lazear).

### Western blot

For whole cell lysates, cells were lysed in radioimmunoprecipitation (RIPA) buffer (150 mM NaCl, 50 mM Tris base, 1% Triton X-100, and 0.5% sodium deoxycholate) supplemented with protease inhibitors for 5 min at room temperature. The cell lysate was incubated on ice for 30 min prior to centrifugation (Eppendorf centrifuge 5424 R, Rotor FA-45-24-11, 13,500 × *g*, 4°C, 20 min). When possible, the total protein concentration of each sample was normalized by bicinchoninic acid (BCA) assay. Protein samples (lysates or affinity-purification [AP] eluates) were resuspended in NuPAGE LDS sample buffer supplemented with tris(2-carboxyethyl)phosphine (TCEP) and boiled at 95°C for 10 min. Samples were run on 7.5%–12.0% polyacrylamide gels for ~1 hour at 150 V and transferred to polyvinylidene fluoride (PVDF) membranes (VWR) for 1 hour at 330 mA on ice. Membranes were then blocked in 5% milk solution for 1 hour prior to overnight incubation in primary antibodies (Table S2) at 4°C. Membranes were washed 3× in Tris-buffered saline with Tween 20 (TBS-T) (150 mM NaCl, 20 mM Tris base, 0.1% Tween 20; Thermo Fisher) and incubated with horseradish peroxidase (HRP) conjugated secondary antibodies in 5% milk for 1 hour at room temperature. Membranes were again washed 3× in TBS-T and 1× in Tris-buffered saline (without Tween 20) prior to Pierce ECL activation (Thermo Fisher). Membranes were imaged using Amersham Imager 600 (GE). Western blot images were analyzed using Fiji. Densitometry was calculated by measuring the band intensity ratio of the experimental band to the loading control band.

### *ANKLE2* CRISPRi knockdown and ZIKV infection

Custom synthetic gRNA were acquired from Sigma and resuspended to 3 µM in TE buffer (10 mM Tris base, 1 mM EDTA, pH 8.0). For Huh7-dCas9 knockdown in 12-well dishes 30 µL of each gRNA was combined with 7 µL TransIT-CRISPR transfection reagent (Sigma) in 363 µL OPTI-MEM (Life Technologies) and complexed at room temperature for 20 min prior to being added to each well (90 nM final gRNA concentration). A total of 1.2 × 10^5^ Huh7-dCas9 cells were then added and grown overnight at 37°C. Additional DMEM was then added at 24 hpt. Viability experiments were done at 72 hpt with ZombieGreen dye (BioLegend, gift of Dr. Scott Dawson) diluted 1:100 in D-PBS and incubated on live cells for 5 min. Ten images were taken for each condition, and total and dead cells were then counted to determine viability. ZIKV replication after *ANKLE2* knockdown was done by removing media from each well at 72 hpt. Two milliliters of fresh DMEM and an appropriate volume of ZIKV stock were then added to each well. Supernatant aliquots were harvested at 0, 18, 24, 48, and 72 hpi and frozen at –80°C.

### Aag2 cell dsRNA knockdown

Gene-specific dsRNA was generated using PCR primers designed to amplify *Ae. aegypti ANKLE2* and containing the T7 promoter sequence. Aag2 cell cDNA generated using the High-Capacity cDNA Reverse Transcription Kit (Applied Biosystems) was used as a template to amplify a 355 bp fragment of the *ANKLE2* transcript for dsRNA synthesis. After PCR amplification, dsRNA was generated via *in vitro* transcription using the MEGAScript RNAi kit (Thermo Fisher). As a non-specific control dsRNA, GFP dsRNA was generated from a GFP-containing plasmid. For Aag2 knockdown in 24-well plates, 500 ng of dsRNA was combined with 1.5 µL of Lipofectamine RNAiMAX (Thermo Fisher), diluted to a total volume of 100 µL in Opti-MEM (Thermo Fisher), and complexed at room temperature for 20 min prior to being added to each well. A total of 2 × 10^5^ cells in 1 mL of culture media was then added to each well. Cells were incubated for 48 hours before RNA extraction or virus infection. Aag2 cells were infected with ZIKV by removing the culture media and adding the appropriate volume of ZIKV diluted in a total of 200 µL of DMEM (no additives). After 1 hour, the virus-containing media was removed and replaced with 1 mL of complete culture media. Supernatant aliquots were harvested at 0, 24, 48, and 72 hpi and frozen at –80°C.

### Plaque assay

Vero cells were grown as a monolayer in six-well dishes overnight. Virus aliquots were thawed on ice then subjected to 10-fold serial dilution. Media were removed from Vero cells, and the monolayer was washed with 1 mL D-PBS. For ZIKV and WNV, 500 µL of each virus dilution was then added and incubated for 1 hour at 37°C with periodic rocking. The virus was then removed, and the cells were overlayed with 3 mL of DMEM with 0.8% methylcellulose (Sigma), 1% FBS, and 1% penicillin/streptomycin (Thomas Scientific) and incubated at 37°C for 4 days. For DENV, cells were infected with 800 µL for 2 hours at 37°C and incubated in DMEM/methylcellulose mixture for 8 days. For YFV, cells were infected with 900 µL for 4 hours at 37°C and incubated in DMEM/methylcellulose mixture for 7 days. Cells were then fixed with 4% formaldehyde (Thermo Fisher) for 30 min at room temperature. Formaldehyde and media were then removed, and cells were stained with 0.23% crystal violet solution (Thermo Fisher) for 30 min. Solution was then removed, and plaques were counted.

### Quantitative RT-qPCR

RNA was harvested using *Quick*-RNA Miniprep kit per manufacturer’s instructions (Zymo). Purified RNA (500 ng) was then used to make cDNA using iScript cDNA synthesis kit (Bio-Rad). After cDNA synthesis, each sample was resuspended to a total volume of 100 µL using RNase-free water. A total of 2 µL of cDNA was then used for each RT-qPCR reaction (with two to three replicate wells for each gene measurement) using LightCycler 480 SYBR Master Mix (Thermo Fisher). Samples were run in a Roche LightCycler 480 II Instrument using relative quantification and temperatures of 95°C (5 s), 55°C (10 s), and 72°C (30 s) for melting, annealing, and elongation, respectively. Quantification of SYBR signal was measured at the end of each elongation step for 40–45 cycles. Changes in gene expression were calculated using the Livak method (2^ΔΔCt^) (64), compared to GAPDH or actin expression as internal controls (56). Primer sequences are listed in Table S4. For tunicamycin experiments, tunicamycin (Thermo Fisher) was dissolved in dimethyl sulfoxide (DMSO) and applied to cells at a final concentration of 10 µg/mL for 12 hours, followed by RNA extraction and RT-qPCR.

### Immunofluorescence microscopy

HEK293T, Huh7, or JEG-3 cells cultured on #1.5 coverslips were fixed with 4% paraformaldehyde (Thermo Fisher) for 15 min at room temperature. Cells were permeabilized with 0.1% Triton X-100 (Integra) for 10 min and blocked with 5% goat serum (Sigma) in PBS-Tween (0.1% Tween 20, Thermo Fisher). Coverslips were incubated with primary antibodies overnight at 4°C. Coverslips were then washed in PBS-Tween and incubated in secondary antibody at room temperature for 1 hour. Nuclei were visualized with Hoechst (Invitrogen). Confocal images were acquired using an Olympus FV1000 Spectral Scan point-scanning confocal fitted to an Olympus IX-81 inverted microscope using a PlanApo ×60/NA1.40 oil immersion lens (Fig. 1) or Zeiss Airyscan LSM980 with Axiocam using a ×63/NA1.40 oil immersion lens (Fig. 2A, M and 4C; Fig. S2I, S3A and B). Laser lines at 405, 488, and 543 nm were employed sequentially for each image using optics and detector stock settings in the “Dye List” portion of the FluoView microscope-controlling software. Other microscopy images (Fig. S2G and data shown in S2, S3, and 4D) were captured using a Nikon Ti2 inverted microscope, CFI PLAN APO LAMBDA ×40 CF160 Plan Apochromat Lambda ×40 objective lens, N.A. 0.95, W.D. 0.17–0.25 mm, F.O.V. 25 mm, DIC, correction collar 0.11–0.23 mm, spring loaded, and using Andor Zyla VSC-08688 camera. All antibodies and dilutions are listed in Table S2. Microscopy images were analyzed using ImageJ (Fiji) software (65). Signal co-localization was quantified using Pearson’s correlation coefficient (*R* value) determined with the “Colocalization 2” analysis tool within Fiji after masking the signal in the entire individual cells across at least five images.

### Semi-automated image analysis

Image analysis was performed using the NIS-Elements AR Software to generate masks on areas of viral protein accumulation. The “General Analysis” function was used for image processing and analysis of the GFP channel. Background correction was performed using a rolling ball background correction method according to the largest sized cluster of viral protein. The “Spot Detection” tool was then used to detect the region’s protein signal. The count and area of aggregates were then automatically quantified for each image. Thresholding was performed using fluorescence intensity as a parameter, whose values depended on the upper and lower bounds of fluorescence intensity of aggregates per image. To minimize the signal-to-noise ratio, a typical size range of 0.3–80.0 µm and a circularity range of 0.2–1.0 were used. For images with larger rearranged membranes that have a non-uniform fluorescence intensity, the “Grow Bright Regions to Intensity” processing tool was used to grow the masked regions to the appropriate size. All other parameter values in the General Analysis tool were kept at their default setting. Manual inspection of images across technical and biological replicates was performed to confirm the accuracy of membrane rearrangement detection.

### ZIKV entry/internalization assay

Cells were seeded into six-well plates 1 day prior to infection. On the day of the infection, cells were pre-cooled on ice for 10 min. Media were removed and the cells were washed with chilled D-PBS. Equal amounts of chilled binding buffer (DMEM pH ~7.4 containing 0.2% bovine serum albumin [BSA], 2 mM MgCl_2_, and 1 mM CaCl_2_) were added to each well. ZIKV PRVABC59 was added at an MOI of 2 and incubated on ice for 90 min. After incubation, the binding buffer was removed, and cells were washed three times with ice cold PBS. Fresh media (DMEM, pH ~7.4, containing 10% FBS) were added to the wells and rapidly warmed up to 37°C. At indicated times, the cells were washed once with D-PBS and lysed in Zymo RNA lysis buffer for RNA purification. Total RNA (500 ng) was converted to cDNA and used for RT-qPCR with either GAPDH or ZIKV primers (see previous). Media supernatant was harvested at later timepoints for PFU quantification using plaque assay.

### Transmission electron microscopy

Cells were mock-infected or infected with ZIKV PRVABC59 for 48 hours prior to collection. Cells were washed with D-PBS and then placed in fixative (2.5% glutaraldehyde, 2% paraformaldehyde, 0.1 M sodium phosphate buffer) for at least 3 hours. Cells were washed with 0.1 M sodium phosphate buffer prior to secondary fixation in 1% osmium tetroxide and 1.5% potassium ferrocyanide for 1 hour. Cells were washed with cold water three times and then serially dehydrated in ethanol (30%, 50%, 70%, 95%, 3 × 100%, 10 min each). Cells were then washed with propylene oxide twice for 10 min each. Half resin (dodecenyl succinic anhydride, Araldite 6005, Epon 812, dibutyl phthalate, benzyldimethylamine) and half propylene oxide were allowed to infiltrate overnight at room temperature. The mixture was then removed and replaced with 100% resin and left to infiltrate for 4 hours. Resin was then replaced with fresh resin and allowed to polymerize at 70°C overnight. Resin blocks were sectioned on Leica EM UC6 ultramicrotome at approximately 100 nm. Sections were collected onto copper grids and dried at 60°C for 20 min. Grids were stained with 4% aqueous uranyl acetate and 0.1% lead citrate in 0.1 N NaOH. Sections were imaged using FEI Talos L120C at 80 kV with a 4k × 4k Ceta camera.

### ANKLE2 and NS4A co-transfection and FLAG affinity purification

For transfection, 5 × 10^6^ HEK293T cells were plated in 10 cm dishes and grown overnight. Transfection was performed by combining 3.5 µg of each corresponding plasmid DNA with 700 µL of serum-free DMEM. Next, 21 µL of PolyJet transfection reagent (SignaGen) was combined with 700 µL serum-free DMEM and added to each plasmid DNA tube. Samples were mixed and incubated at room temperature for 15 min prior to addition to cells. Cells were then grown for an additional 24 hours. Transfection efficiency was confirmed using a GFP-encoding plasmid. Media were then removed from each plate. To dissociate cells, 5 mL of D-PBS supplemented with 10 mM EDTA was added and allowed to incubate for several minutes. Cells were resuspended in 5 mL of D-PBS and transferred to 15 mL conical tubes prior to centrifugation at 94 × *g*, 4°C for 5 min (Eppendorf centrifuge 5810 R, Rotor S-4–104). Cell pellets were washed with 5 mL D-PBS and centrifugation was repeated. Supernatant was removed and pellets were then resuspended in 1 mL IP buffer (50 mM Tris base, 150 mM NaCl, 0.5 M EDTA, pH 7.4) with Pierce protease inhibitor tablets (Thermo Scientific) supplemented with 0.5% NP-40 Substitute (Igepal CA-630, Affymetrix). Cells were lysed for 30 min at 4°C, and lysate was then centrifugated at 845 × *g*, 4°C, for 20 min (Eppendorf centrifuge 5424 R, Rotor FA-45–24-11). A portion of each lysate (60–100 µL) was collected, normalized by BCA assay (Thermo Scientific), and saved for Western blot analysis. Remaining lysate was added to 40 µL of magnetic FLAG beads (Sigma) and incubated overnight at 4°C with gentle rotation. Beads were then washed four times with 1 mL IP buffer with 0.05% NP-40 and once with 1 mL IP buffer without NP-40. Beads were then incubated in 40 µL of 100 ng/mL FLAG peptide (APExBIO) at 211 × *g* for 1 hour at room temperature (Eppendorf ThermoMixerC). The eluate was then removed. The eluate and lysate were resuspended in NuPAGE LDS sample buffer and bond-breaker TCEP (Thermo Scientific) according to the manufacturer’s recommendation. Samples were boiled for 10 min at 95°C prior to evaluation by Western blot (below).

### Statistical analysis

Statistical analysis and plotting were performed using GraphPad Prism software (version 6.0; GraphPad Software Inc., La Jolla, CA, USA). Error bars represent standard deviations. Data were considered statistically significant when a *P* value of <0.05 was determined by Student’s *t*-test or one-way analysis of variance with noted multiple comparison test.

## SUPPLEMENTAL MATERIAL

Supplemental figures and tables mbio.02683-24-s0001.docx Supplemental figures and tables.

Table S1 mbio.02683-24-s0002.xlsx CRISPR mutagenesis sequencing.

ASM does not own the copyrights to Supplemental Material that may be linked to, or accessed through, an article. The authors have granted ASM a non-exclusive, world-wide license to publish the Supplemental Material files. Please contact the corresponding author directly for reuse.
