# Supplementary material for: Microcephaly protein ANKLE2 promotes Zika virus replication
Source: mBio. 2025 Jan 13;16(2):e02683-24. doi: 10.1128/mbio.02683-24 (PMC11796389; doi:10.1128/mbio.02683-24)
Supplement: Supplemental figures and tables [file mbio.02683-24-s0001.docx]

**Supplementary Figures**


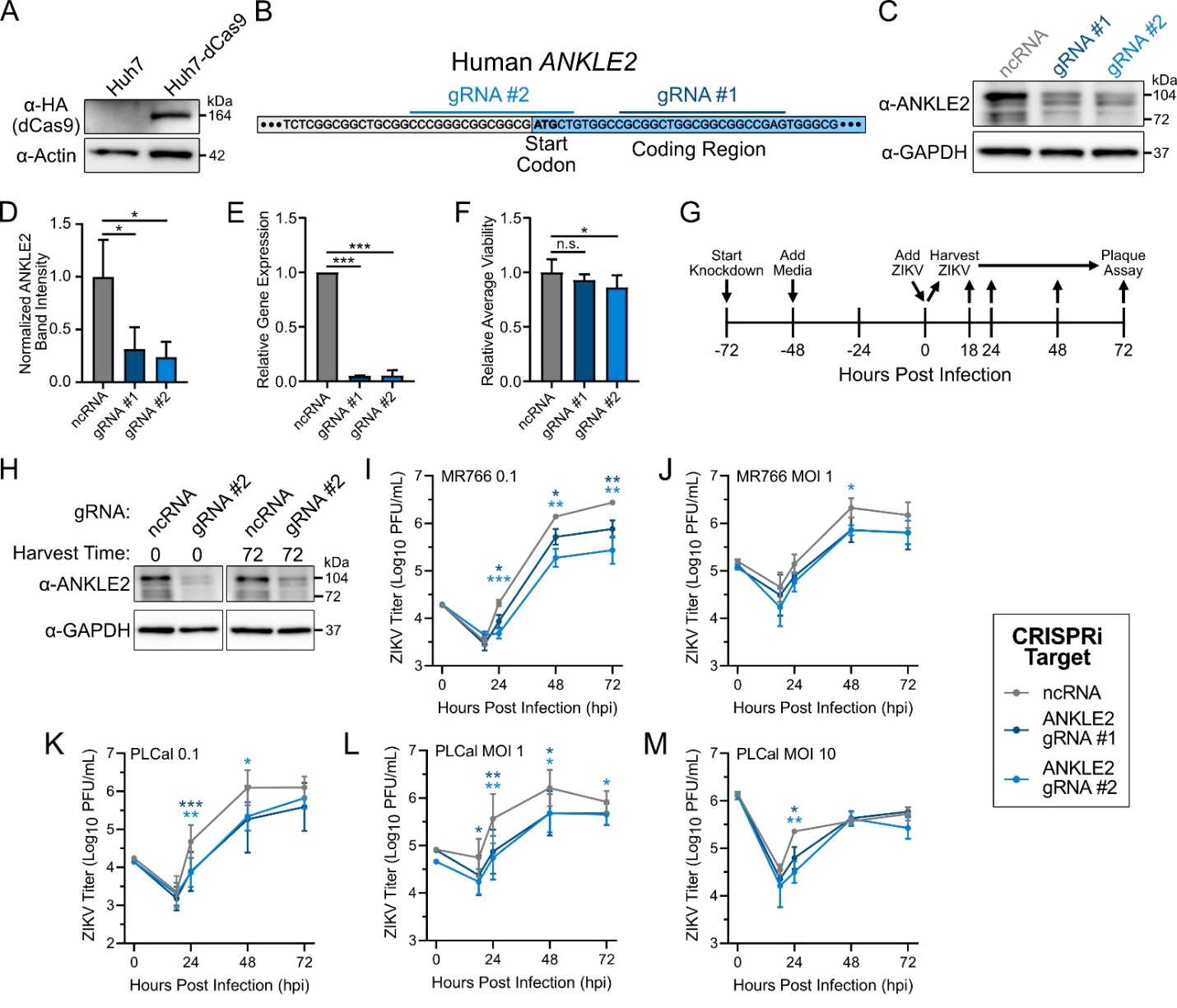
**Fig. S1: CRISPRi knockdown of ANKLE2 in Huh7-dCas9 cells reduces ZIKV replication.** (A) Western blot of dCas9-HA in Huh7 cells used for CRISPRi experiments. (B) Schematic of ANKLE2 and the targeting guide RNAs (gRNA) used to knockdown ANKLE2 expression. (C) Western blot to evaluate ANKLE2 expression after CRISPRi knockdown. Knockdown of known ANKLE2 isoforms can be observed at 76 and 72 kdA. A known background band associated with this antibody at ~96kDa is visible and unchanged. (D) Densitometry of ANKLE2 band intensity relative to its respective loading control and then normalized to ncRNA. Error bars represent the standard deviation between 3 biological replicates. (E) ANKLE2 gene expression after knockdown was quantified by RT-qPCR. Error bars represent the standard deviation between 3 biological replicates. (F) Cell viability after knockdown was evaluated using ZombieGreen dye. Data represents the average cell viability across 10 images for each condition. (G) Schematic for 72-hour CRISPRi knockdown, followed by 72-hour ZIKV infection. Supernatant was harvested from infected cells at 0, 18, 24, 48, and 72 hours post infection and assessed by plaque assay. (H) Western blot validation that ANKLE2 knockdown persists for additional 72 hours after removal of transfection reagents. (I-M) After a 72-hour knockdown, cells were infected with ZIKV MR766 or PLCal at designated MOI. Virus titers were determined using plaque assay. All error bars represent the standard deviation. (I, J, M) Three technical replicates. Student’s unpaired two-tailed t-test. (K, L) Five biological replicates. Student’s paired two-tailed t-test. All statistical tests compared to ncRNA condition, n.s., not significant, * p < 0.05, ** p < 0.01, *** p < 0.001.


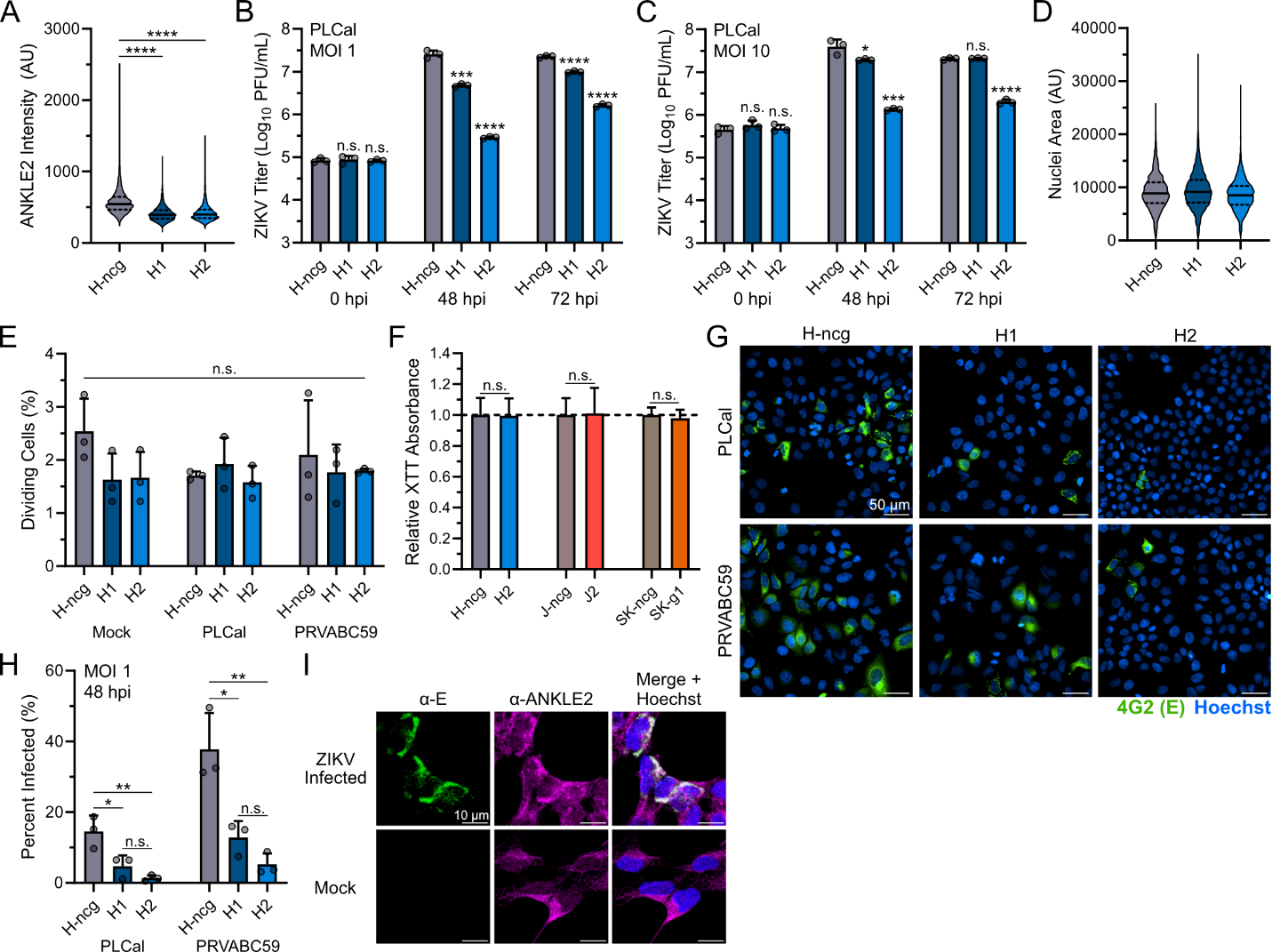


**Fig. S2: Characterization of ANKLE2 knockout cells.** (A) ANKLE2 fluorescence intensity was measured across three biological replicates, n = ~1500 - 2100 cells per condition. (B-C) Virus titer was determined by plaque assay following infection of control and ANKLE2 KO cells. All statistical comparisons are made to negative control (H-ncg) cells. (D) Nuclei area was determined using cellpose masking. Three biological replicates, n = ~2600 - 3300 cells per condition. (E) Blinded images were assessed for cells visibly in any stage of mitosis. Grey circles represent the average value of each technical replicate (at least 10 images per replicate). (F) Cell viability was measured for control and ANKLE2 KO cells using a XTT assay. (G) Representative images of cells infected with ZIKV (PLCal or PRVABC59) at MOI 1 for 48 hours used to determine percent of cells infected. (H) ZIKV infectivity measured by immunofluorescence microscopy of flavivirus E protein detected by 4G2. Image identities were blinded prior to counting infected cells. Three replicate infections, at least 10 images per replicate, n = ~2800 – 5600 total cells per condition. (I) Immunofluorescence microscopy of SK-N-SH cells infected with ZIKV PRVABC59 at MOI 1 for 48 hours. Data representative of either three technical replicates (B-C) or three biological replicates (A, D-G, I). Grey dots indicate individual replicates. All statistical tests compared to ncgRNA condition, n.s., not significant, * p < 0.05, ** p < 0.01, *** p < 0.001, **** p < 0.0001. One-way ANOVA with Dunnett's multiple comparisons when appropriate (A, E) or student’s T-test (B, C, F, and H).


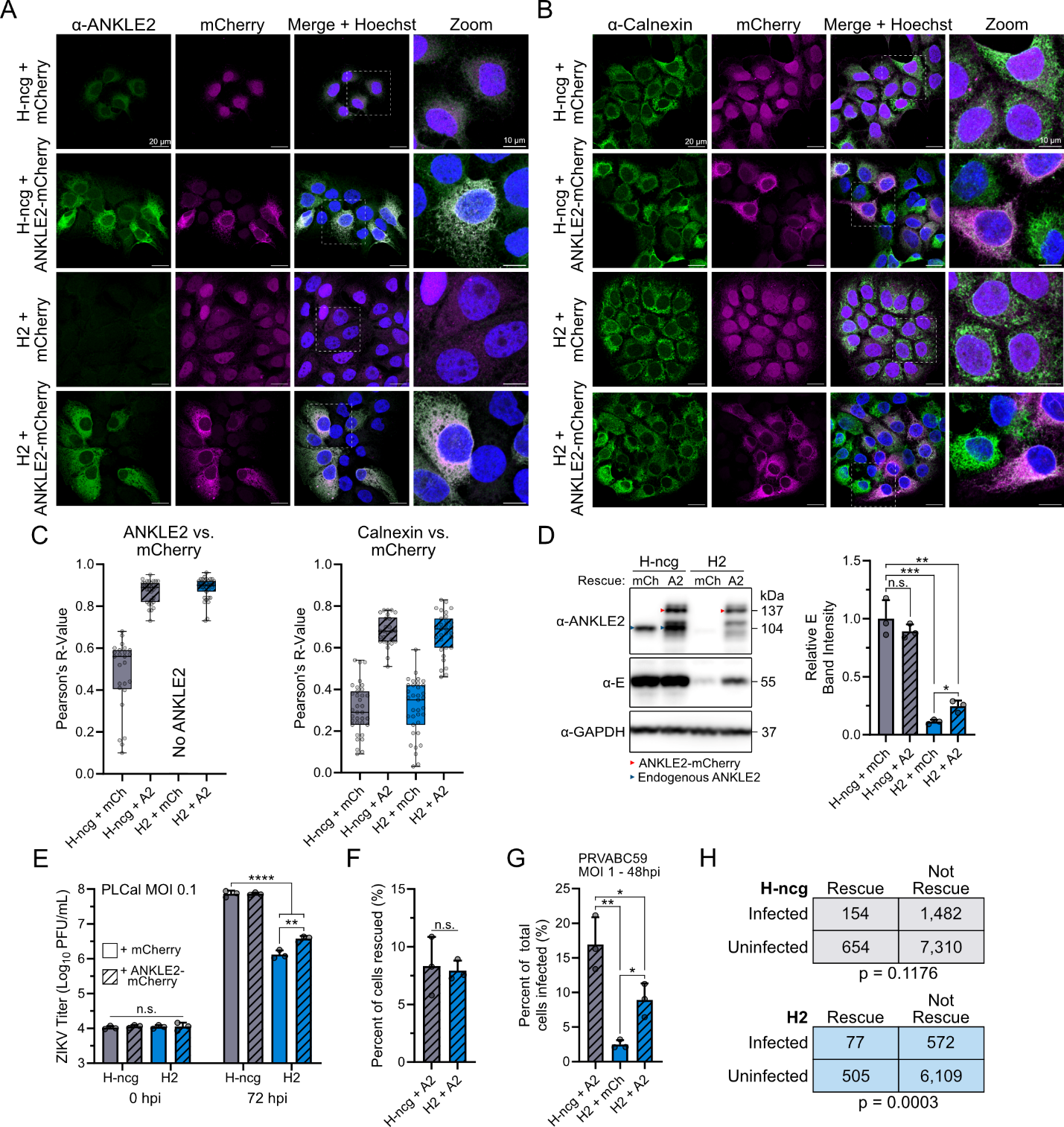


**Fig. S3: Restoration of ANKLE2 to Huh7 knockout cells partially rescues virus replication phenotype.** H-ncg control or H2 KO cells were transduced with lentivirus to express mCherry (mCh) or ANKLE2-mCherry-3xFLAG (A2). (A-B) Confocal microscopy for ANKLE2 (A) or ER marker Calnexin (B). (C) Pearson's Correlation was used to measure colocalization between mCherry and ANKLE2 or Calnexin. Grey dots represent individual cells, n = 25-35 cells. Scale bars = 20 µm or 10 µm (zoom). (D) Western blot of cells infected with ZIKV PLCal at MOI 0.1 for 72 hours. ZIKV E protein levels were quantified by densitometry. (E) Virus titers were measured using plaque assay. (F-G) Cells were infected with ZIKV PRVABC59 at MOI 1 for 48 hours and immunostained for FLAG and ZIKV E. Fluorescent images were acquired (at least 10 per replicate) and identities were blinded prior to counting the number of rescue and infected cells. Grey dots represent rescue or infection rate from all cells within each of the three technical replicates (n = 2100-3400 cells per replicate). Student's two-tailed t-test, n.s., not significant, * p < 0.05, ** p < 0.01, *** p < 0.001, **** p < 0.0001. (H) Fisher's exact test was used to evaluate probability of infection based on ANKLE2-mCherry expression in control or KO cells based on (F-G).


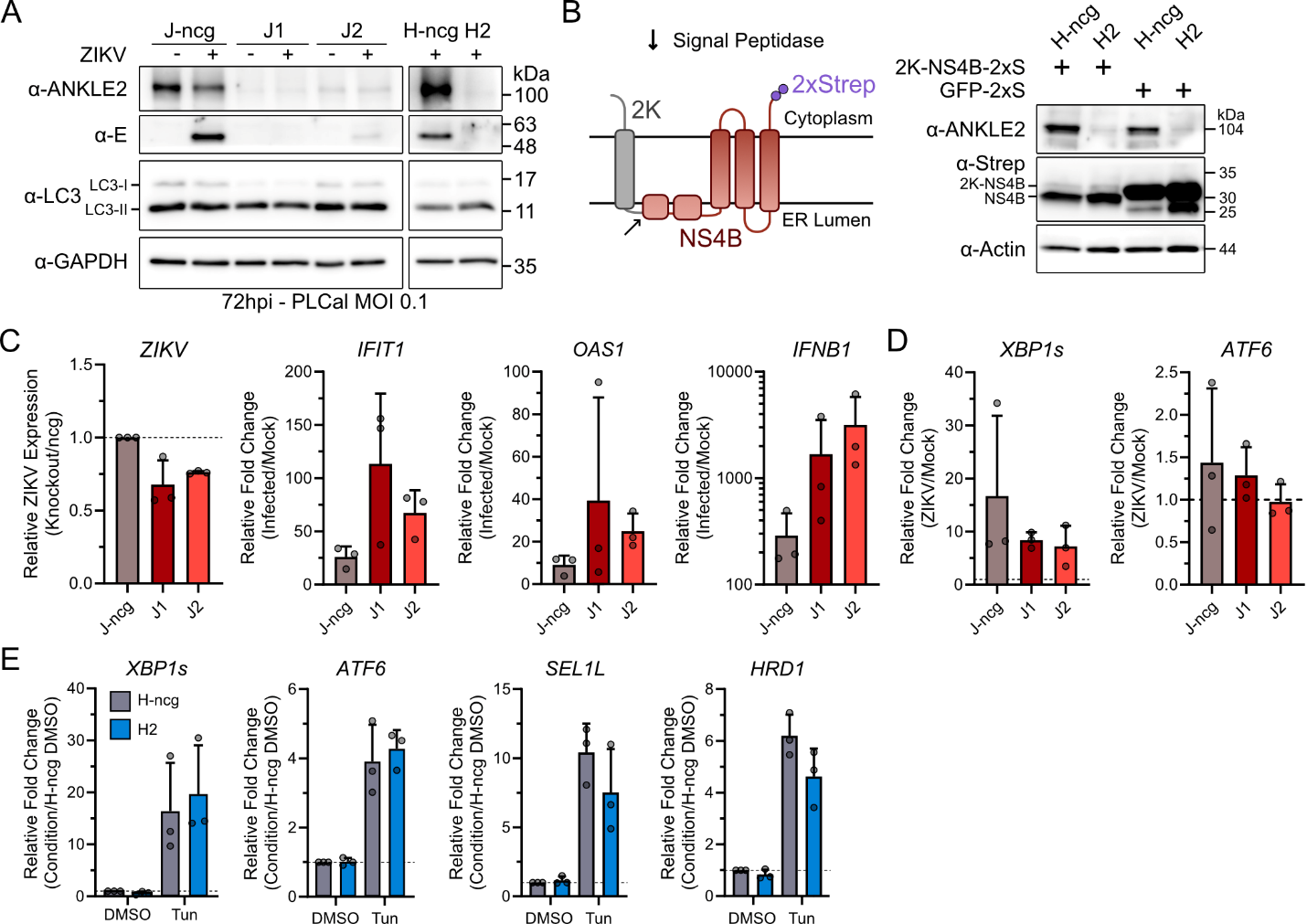


**Fig. S4: Evaluation of autophagy and ER processes in ANKLE2 knockout cells.** (A) Western blot assessing autophagy marker LC3B in ANKLE2 KO JEG-3 and Huh7 cells after ZIKV infection. (B) Huh7 cells were transfected with plasmid expressing ZIKV 2K-NS4B-2xStrep to assess signal peptidase cleavage efficiency. GFP-2xStrep was transfected as a control. (C-D) RT-qPCR of ER-stress genes from JEG-3 cells infected with ZIKV PRVABC59 at MOI 5 for 24 hours. (E) Huh7 cells were treated with 1% DMSO or 10 µg/mL tunicamycin (Tun) in DMSO for 12 hours prior to gene expression analysis by RT-qPCR. Grey dots represent individual biological replicates.


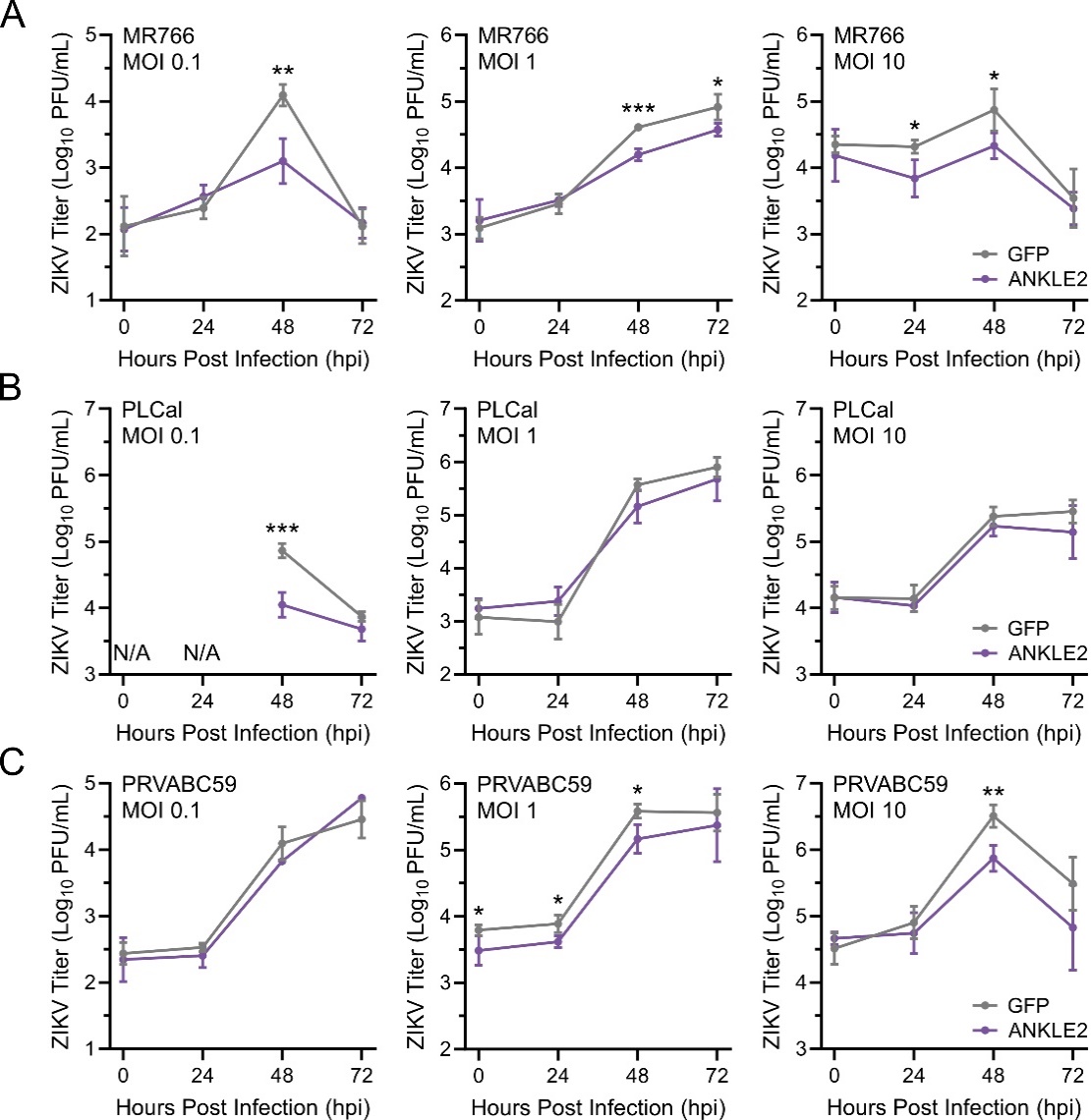


**Fig. S5: Full time-courses of dsRNA ANKLE2 silencing in mosquito Aag2 cells.** (A-C) After dsRNA transfection, Aag2 cells were infected with noted ZIKV strain at either MOI 0.1, 1, or 10. Viral supernatant was harvested and virus titers were measured by plaque assay. Student's unpaired t-test, * p < 0.05, ** p < 0.01, *** p < 0.001. Any timepoints without stars are not statistically significant (p > 0.05).

**Supplementary Table 1: CRISPR Mutagenesis Sequencing**

Included as Excel file.

**Supplementary Table 2: Antibodies**

| Antibody | Host Species | Dilution Used | Supplier (Catalog #) | RRID |
| --- | --- | --- | --- | --- |
| GAPDH | Mouse | 1:1000 (WB) | Fisher (PIMA515738) | AB_2537652 |
| FLAG-M2 | Mouse | 1:200 (IF), 1:1000 (WB) | MilliporeSigma (F1804) | AB_262044 |
| Strep | Mouse | 1:1000 (WB) | Qiagen (34850) | AB_2810987 |
| 4G2 (Orthoflavivirus E) | Mouse | 1:250 (IF) | ATCC (HB-112) | CVCL_J890 |
| SERCA2 | Mouse | 1:100 (IF) | Fisher (MA3919) | AB_325502 |
| FLAG Tag | Rabbit | 1:400 (IF) | Cell Signaling (14793) | AB_2572291 |
| ANKLE2 | Rabbit | 1:80 (IF) | Atlas Antibodies (HPA074838) | N/A |
| ANKLE2 | Rabbit | 1:1000 (WB) | Atlas Antibodies (HPA003602) | AB_1858349 |
| ANKLE2 | Rabbit | 1:1000 (WB) | Bethyl Labs (A302-966A-M) | AB_2780882 |
| ZIKV Envelope (E) | Rabbit | 1:1000 (WB) | GeneTex (GTX637298) | N/A |
| ZIKV NS4A | Rabbit | 1:1000 (IF, WB) | GeneTex (GTX133704) | AB_2887067 |
| ZIKV Capsid | Rabbit | 1:1000 (WB) | GeneTex (GTX133317) | AB_2756861 |
| Actin | Rabbit | 1:5000 (WB) | Sigma (A2066) | AB_476693 |
| Anti-Mouse IgG-HRP | Rabbit | 1:5000 (WB) | SouthernBiotech (6170-05) | AB_2796243 |
| Calnexin | Rabbit | 1:250 (IF) | Proteintech (10427-2-AP)  Gift from Dr. Jodi Nunnari | AB_2069033 |
| LC3B (D11) | Rabbit | 1:1000 (WB) | Cell Signaling (#3868) | AB_2137707 |
| HA | Rat | 1:2000 (WB) | MilliporeSigma (3F10)  Gift from Dr. Joanna Chiu | AB_2314622 |
| Anti-Rabbit IgG-HRP | Goat | 1:5000 (WB) | SouthernBiotech (4030-05) | AB_2687483 |
| Anti-Mouse AlexaFlour-488 | Goat | 1:1000 (IF) | Invitrogen (A28175) | AB_2536161 |
| Anti-Mouse AlexaFlour-555 | Goat | 1:1000 (IF) | Invitrogen (A21422) | AB_2535844 |
| Anti-Rabbit AlexaFlour-488 | Goat | 1:1000 (IF) | Invitrogen (A11008) | AB_143165 |
| Anti-Rabbit AlexaFlour-555 | Goat | 1:1000 (IF) | Invitrogen (A27039) | AB_2536100 |
| Anti-Mouse AlexaFlour-680 | Goat | 1:1000 (IF) | Invitrogen (A21057) | AB_2535723 |

WB = Western blot, IF = Immunofluorescence

**Supplementary Table 3: Sequences**

| Sequence | Accession Number | Relevant Figure(s) |
| --- | --- | --- |
| Human ANKLE2 | NM_015114.3 | 1, 2, 6, S3 |
| Human ANKLE1 | NM_152363.6 | 1 |
| eGFP | UDY80669.1 | 1, S4 |
| mCherry | AY678264.1 | S3, 2 |
| Mosquito ANKLE2 | XM_021856854.1 | 5, S5 |
| Zika virus (ZIKV) (PRVABC59) | MK713748.1 | S4B, 4B-D, 6 |
| Dengue virus serotype 2 (DENV2) | NC_001474.2 | 6 |
| Yellow Fever virus (YFV) | KF769016.1 | 6 |
| West Nile virus (WNV) | DQ211652.1 | 6 |
| Japanese encephalitis virus (JEV) | NC_001437.1 | 6 |

**Supplementary Table 4: Oligonucleotides**

| Oligo Name | Sequence | Application |
| --- | --- | --- |
| ANKLE2 CRISPRi gRNA #1 | GCGGCTGGCGGCGGCCGAGT | CRISPRi sygRNA |
| ANKLE2 CRISPRi gRNA #2 | GCCGGGCGGCGGCGATGCTG | CRISPRi sygRNA |
| Negative CRISPRi gRNA | Proprietary sequence from Millipore Sigma | CRISPRi sygRNA |
| ANKLE2 CRISPR Forward Oligo | CACCGCCGCGCTTGGCGGAGGAACT | CRISPR gRNA |
| ANKLE2 CRISPR Reverse Oligo | AAACAGTTCCTCCGCCAAGCGCGG | CRISPR gRNA |
| Negative CRISPR gRNA Forward Oligo | CACCGACCCTCCGAATCGTAACGGA | CRISPR gRNA |
| Negative CRISPR gRNA Reverse Oligo | AAACTCCGTTACGATTCGGAGGGTC | CRISPR gRNA |
| ANKLE2 Sequencing Forward w/ Adapter | *ACACTCTTTCCCTACACGACGCTCTTCCGATCT*GTGCTGCTGATCGCTGTG | Sequencing ANKLE2 KOs  *(with Illumina Adapter)* |
| ANKLE2 Sequencing Reverse w/ Adapter | *GACTGGAGTTCAGACGTGTGCTCTTCCGATCT*GTGGTAGAAAGAAGACAGCC | Sequencing ANKLE2 KOs  *(with Illumina Adapter)* |
| GAPDH Forward | ACATCGCTCAGACACCATG | RT-qPCR Control |
| GAPDH Reverse | TGTAGTTGAGGTCAATGAAGGG | RT-qPCR Control |
| ANKLE2 Forward | AAAGAGAACCAGGCTTCCATC | RT-qPCR Analysis (Human) |
| ANKLE2 Reverse | CACGTTGACTACATCTGCATTTC | RT-qPCR Analysis (Human) |
| ZIKV Forward | CGCCACCAAGATGAACTGATTG | RT-qPCR Analysis |
| ZIKV Reverse | CATCCATTCTCCCTTTCCATGGAT | RT-qPCR Analysis |
| IFNB1 Forward | TGCTCTCCTGTTGTGCTTCTC | RT-qPCR Analysis |
| IFNB1 Reverse | TAGATGGTCAATGCGGCGTC | RT-qPCR Analysis |
| IFIT1 Forward | CTTGGGTTCGTCTACAAATTGG | RT-qPCR Analysis |
| IFIT1 Reverse | AAAGTGGCTGATATCTGGGTG | RT-qPCR Analysis |
| OAS1 Forward | ATAAAAGCAAACAGGTCTGG | RT-qPCR Analysis |
| OAS1 Reverse | TCTGGCAAGAGATAGTCTTC | RT-qPCR Analysis |
| XBP1s Forward | GCTGAGTCCGCAGCAGGT | RT-qPCR Analysis |
| XBP1s Reverse | CTGGGTCCAAGTTGTCCAGAAT | RT-qPCR Analysis |
| ATF6 Forward | TCCAGCAGCACCCAAGACTC | RT-qPCR Analysis |
| ATF6 Reverse | CCAGCAACAGCAAGGACTG | RT-qPCR Analysis |
| SEL1L Forward | ATCTCCAAAAGGCAGCAAGC | RT-qPCR Analysis |
| SEL1L Reverse | TGGGAGAGCCTTCCTCAGTC | RT-qPCR Analysis |
| HRD1 Forward | CTTCACCGTTTTTCGGGATGA | RT-qPCR Analysis |
| HRD1 Reverse | CCAGGAGGAACATAAGAGAGACA | RT-qPCR Analysis |
| ANKLE2 dsRNA Target Forward | *TAATACGACTCACTATAGGG*GCTGGAAATCAAAGCCTACG | Aag2 dsRNA Knockdown  *(with T7 Promoter)* |
| ANKLE2 dsRNA Target Reverse | *TAATACGACTCACTATAGGG*TTTCTCGTCCAGTTGTCGCT | Aag2 dsRNA Knockdown  *(with T7 Promoter)* |
| GFP dsRNA Target Forward | *TAATACGACTCACTATAGGG*ATGGTGAGCAAGGGCGAGGAGCTGTTC | Aag2 dsRNA Knockdown  *(with T7 Promoter)* |
| GFP dsRNA Target Reverse | *TAATACGACTCACTATAGGG*CTGGGTGCTCAGGTAGTGGTTGTCGGGC | Aag2 dsRNA Knockdown  *(with T7 Promoter)* |
| ANKLE2 Forward | CCTCCAGAACTTCCTCGATTTC | RT-qPCR Analysis (*Aedes*) |
| ANKLE2 Reverse | CGGAGGAGTTCGTCTGATTATTT | RT-qPCR Analysis (*Aedes*) |
| GFP Forward #1 | TAAAGGCCGCCATGGTGAGCAA | Gibson Assembly preparation of GFP-APEX2-3xFLAG |
| GFP Reverse #1 | CGCTCTTGTACAGCTCGTCCAT | Gibson Assembly preparation of GFP-APEX2-3xFLAG |
| GFP Forward #2 | CTTATACCAACTTTCCGTACCACTTCCTACCCTCGTAAAGGCCGCCATGGTGAGCA | Gibson Assembly of GFP-APEX2-3xFLAG |
| GFP Reverse #2 | CAGATCCACCTCCTGAACCACCTCCGCTACCGCCACCGCTCTTGTACAGCTCGTCCATGC | Gibson Assembly of GFP-APEX2-3xFLAG |
| ANKLE2 Forward #1 | TAAAGGCCGCCATGCTGTGGCCGCGG | Gibson Assembly preparation of ANKLE2-APEX2-3xFLAG |
| ANKLE2 Reverse #1 | GCTCAGGGCGGCAAGCTCAGCCAGG | Gibson Assembly preparation of ANKLE2-APEX2-3xFLAG |
| ANKLE2 Forward #2 | ACTTCCTACCCTCGTAAAGGCCGCCATGCTG | Gibson Assembly of ANKLE2-APEX2-3xFLAG |
| ANKLE2 Reverse #2 | CCGCTACCGCCACCGCTCAGGGCGGCAAG | Gibson Assembly of ANKLE2-APEX2-3xFLAG |
| ANKLE1 Forward #1 | GCCGCCATGTGTTCCGAAGCCC | Gibson Assembly preparation of ANKLE1-APEX2-3xFLAG |
| ANKLE1 Reverse #1 | CGCTTCCACGCGCTTGAATATCT | Gibson Assembly preparation of ANKLE1-APEX2-3xFLAG |
| ANKLE1 Forward #2 | ACTTCCTACCCTCGTAAAGGCCGCCATGTGT | Gibson Assembly of ANKLE1-APEX2-3xFLAG |
| ANKLE1 Reverse #2 | TCCGCTACCGCCACCGCTTCCACGCG | Gibson Assembly of ANKLE1-APEX2-3xFLAG |
| pcDNA Upstream | TGGGAGTTTGTTTTGGAACCA | Sequencing of pcDNA constructs |
| pcDNA Downstream | CAGATGGCTGGCAACTAGAAG | Sequencing of pcDNA constructs |
| Mycoplasma Forward #1 | TGCACCATCTGTCACTCTGTTAACCTC | For *Mycoplasma* spp*.* detection |
| Mycoplasma Reverse #1 | GGGAGCAAACAGGATTAGATACCCT | For *Mycoplasma* spp*.* detection |
| Mycoplasma Forward #2 | GGCGAATGGGTGAGTAACACG | For *Mycoplasma* spp*.* detection |
| Mycoplasma Reverse #2 | CGGATAACGCTTGCGACCTATG | For *Mycoplasma* spp*.* detection |
